# Robotic Actuation‐Mediated Quantitative Mechanogenetics for Noninvasive and On‐Demand Cancer Therapy

**DOI:** 10.1002/advs.202401611

**Published:** 2024-03-21

**Authors:** Yangyi Liu, Jingjing Li, Yi Zhang, Fan Wang, Juanjuan Su, Chao Ma, Shuyi Zhang, Yanan Du, Chunhai Fan, Hongjie Zhang, Kai Liu

**Affiliations:** ^1^ Center of Materials Science and Optoelectronics Engineering College of Materials Science and Optoelectronic Technology University of Chinese Academy of Sciences Beijing 100049 China; ^2^ Engineering Research Center of Advanced Rare Earth Materials (Ministry of Education) Department of Chemistry Tsinghua University Beijing 100084 China; ^3^ State Key Laboratory of Rare Earth Resource Utilization Changchun Institute of Applied Chemistry Chinese Academy of Sciences Changchun 130022 China; ^4^ School of Pharmaceutical Sciences Tsinghua University Beijing 100084 China; ^5^ Department of Biomedical Engineering School of Medicine Tsinghua‐Peking Center for Life Sciences Tsinghua University Beijing 100084 China; ^6^ Xiangfu Laboratory Jiaxing 314102 China; ^7^ School of Chemistry and Chemical Engineering New Cornerstone Science Laboratory Frontiers Science Center for Transformative Molecules Zhangjiang Institute for Advanced Study and National Center for Translational Medicine Shanghai Jiao Tong University Shanghai 200240 China

**Keywords:** biomedical engineering, chemical biology, mechanogenetics, precise treatment, tumor therapy

## Abstract

Cell mechanotransduction signals are important targets for physical therapy. However, current physiotherapy heavily relies on ultrasound, which is generated by high‐power equipment or amplified by auxiliary drugs, potentially causing undesired side effects. To address current limitations, a robotic actuation‐mediated therapy is developed that utilizes gentle mechanical loads to activate mechanosensitive ion channels. The resulting calcium influx precisely regulated the expression of recombinant tumor suppressor protein and death‐associated protein kinase, leading to programmed apoptosis of cancer cell line through caspase‐dependent pathway. In stark contrast to traditional gene therapy, the complete elimination of early‐ and middle‐stage tumors (volume ≤ 100 mm^3^) and significant growth inhibition of late‐stage tumor (500 mm^3^) are realized in tumor‐bearing mice by transfecting mechanogenetic circuits and treating daily with quantitative robotic actuation in a form of 5 min treatment over the course of 14 days. Thus, this massage‐derived therapy represents a quantitative strategy for cancer treatment.

## Graphical abstract

The robotic actuation‐mediated mechanogenetic therapy is constructed for on‐demand cancer treatment. Gentle mechanical pressing stimulus initiated the expression of anti‐tumor proteins in cancer cells, leading to programmed tumor apoptosis through caspase‐dependent pathway. By this strategy, the complete elimination of early‐ and middle‐stage tumors and significant growth inhibition of late‐stage tumor are realized in tumor‐bearing mice models.

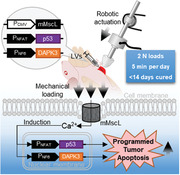

## 1 Introduction

Mechanical signals sensed by cells orchestrate cellular behavior at both local and systemic levels within their microenvironment.^[^ ^1^ ^]^ This ability is critical for guiding cell fate and maintaining homeostasis.^[^ ^2^ ^]^ Consequently, the mechanotransduction signals have become therapeutic targets for physical therapy.^[^ ^3^ ^]^ Recently, physical therapy taking advantage of ultrasound technique emerges as an attractive tool for stimulating cellular activities.^[^ ^4^ ^]^ However, the mechanical stimulations of ultrasound are produced by high‐power equipment^[^ ^5^ ^]^ and amplified by nanoparticles^[^ ^6^ ^]^ or small molecule compound,^[^ ^7^ ^]^ often leading to subsequent thermal injuries derived from energy accumulation in the tissue^[^ ^8^ ^]^ as well as other undesired side effects.^[^ ^9^ ^]^ In contrast, the robotic actuation that is a soft and convenient strategy,^[^ ^10^ ^]^ simplifies energy conversion, and applies pressure directly to the cell zones of interest, thus overcoming the limitations of ultrasound therapy and greatly elevating patient compliance.

Robotic actuation‐mediated therapy, inspired by massage, is a practical technique for relieving muscle stiffness and tissue pain that is commonly used in daily life.^[^ ^11^ ^]^ However, there remains a significant challenge in incorporating mechanical stimuli to induce the endogenous gene expression in cells.^[^ ^12^ ^]^ Consequently, robotic actuation‐mediated therapy nowadays lacks of precision and remains a palliative physical treatment.^[^ ^13^ ^]^ It is essential to establish a mechanogenetic pathway to leverage the benefits of robotic actuation‐mediated therapy for accurate treatment. Specifically, gene expression is regulated by manipulating cellular signaling and behavior through mechanical force, offering a reversible process with precise spatiotemporal resolution. Therefore, we explore the possibility of mechanical loads as a quantitatively controlled, safe, and convenient model for disease treatment.

**Figure 1:**
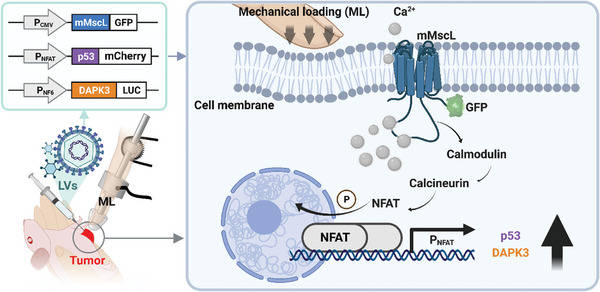
Mechanical loading (ML) induced gene expression system for oncotherapy. The heterologous mutant mechanosensitive channel (mMscL) that was transferred via lentiviral vectors (LVs) into mammalian cells has the ability to transport calcium ions under the control of ML. Calcium influx activates the calmodulin/calcineurin pathway, which leads to dephosphorylation of a nuclear factor of activated T cells (NFAT) and its translocation to the nucleus, where it activates the NFAT‐sensitive promoter (P_NFAT_) and triggers downstream cancer suppressor gene expression. All of the proteins transfected by LVs can be detected by the bio‐fluorescence from reporter protein, such as green fluorescent protein (GFP) for mMscL, red fluorescent protein mCherry for tumor suppressor p53 and luciferase protein (LUC) for death associated protein kinase 3 (DAPK3). This figure was created with reference to pictures in BioRender.com.

In this study, we developed a robotic actuation‐mediated mechanogenetic system for the regulation of cell activities, as depicted in **Figure** **1**. This system consists of two essential modules, the robotic actuation device and the engineered mechanogenetic circuits. The mechanosensitive channel (MscL) as a mechanical loading sensor was applied to generate calcium influx that activates a nuclear factor of activated T cells (NFAT), thus initiating expression of downstream target genes, tumor suppressor p53, and death associated protein kinase 3 (DAPK3), within the mechanogenetic circuits. After transfection into the mammalian breast tumor cell MCF7 in vitro and the tumor‐bearing Balb/c mice in vivo, the mechanogenetic circuits showed excellent anti‐tumor efficacy, with complete regression of tumors with a volume of 100 mm^3^ level, and significant growth inhibition of 500 mm^3^‐level tumors. This robotic‐controlled synergistic expression of two antitumor genes surpassed direct expression, offering an adaptable therapeutic approach aligned with tumor progression. Therefore, this robotic actuation‐mediated mechanogenetic therapy provides a powerful tool, which allows a non‐invasive, on‐demand, and quantitative cancer treatment.

## 2 Results

### 2.1 Mechanical Loading‐Induced Ca^2+^ Influx in Mammalian Cells for Expressing mMscL

**Figure 2:**
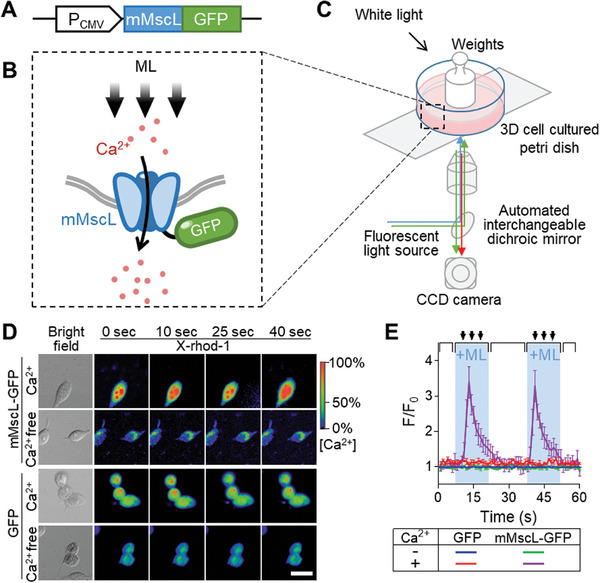
The construction and detection of mechanical loading‐induced Ca^2+^ influx in mammalian cells expressing mMscL. A) Schematic representation of the mMscL gene fused to an enhanced GFP coding gene for heterologous expression test. B) Diagram of mechanical force sensing in engineered mammalian cells. At resting state, the artificial Ca^2+^ channel is closed and cannot lead to membrane depolarization. When cell membrane was deformed by mechanical loading, the mMscL channel opened up and mediated Ca^2+^ influx. C) Diagram of an integrated system for ML‐induced fluorescence imaging. The ML was applied using weights and the cells were adhesively cultured on the glass bottle of the 3D cell cultured Petri dish. The CCD camera captured fluorescence signals of GFP or X‐rhod‐1 with two emission filters (488 and 561 nm) controlled by a filter changer. D) Time courses of the relative X‐rhod‐1 fluorescence induced by ML. Scale bar: 20 µm. E) Statistical analysis of amplitude (F/F0) of X‐rhod‐1. Black arrow and blue background indicate the weight was set down for mechanical loading. Error bars indicate SEM, n = 3.

MscL, as a mechanosensitive channel in bacteria, can sense changes in membrane tension and open up for calcium influx under external mechanical loading (ML).^[^ ^14^ ^]^ Here, we adopted a mutant type of MscL, MscL‐I92L (hereafter referred to as mMscL), to build stimulation‐induced sensors in mammalian cells.^[^ ^15^ ^]^ First, we tested the expression level and location of the heterologous mMscL channel in mammalian cells, including murine mammary carcinoma cell line 4T1, human breast cancer cell line MCF7, and human embryonic kidney cell line (293T. The gene encoding sequences of the mMscL from *E. coli* were codon‐optimized and cloned after the cytomegalovirus overexpression promoter (P_CMV_) (**Figure** **2**A). In addition, one green fluorescent protein (GFP) gene was fused to the 3′‐end of mMscL in order to indicate the location of the mMscL channel. The expressing plasmid of mMscL‐GFP was transfected into mammalian cell lines by transient chemical method. The expression of mMscL‐GFP can be observed by fluorescence microscopy (Figure 2B). As shown in Figure S1 (Supporting Information), the cell lines containing mMscL‐GFP expression cassette showed obvious fluorescent signals specifically located at the cell membrane, whereas the control group cells expressing free GFP protein exhibited fluorescent signals in the whole cytoplasm. The results clearly demonstrated that mMscL, the mechanosensitive calcium ion channel originated from *E. coli*, can be efficiently expressed and correctly localize in mammalian cells. Besides, there was no obvious selectivity for the type of expressing cells.

We then examined the possibility of ML to activate the mMscL channel for eliciting Ca^2+^ influx in 293T cells. The mMscL‐GFP expression cassette was used for the transfection. To simultaneously observe the Ca^2+^ influx, a fluorescent indicator X‐rhod‐1 was cultured with cells.^[^ ^16^ ^]^ We examined the intracellular Ca^2+^ levels with a ML‐fluorescence imaging system (Figure 2C). The fluorescence intensity at 561 nm indicated the movement of calcium ions into cells. Specifically, the ML was applied by setting down the weight at 7.5 s. Then the weight was lifted up or set down every 15 s and the ML was repeated twice. Time‐course of the red fluorescence of X‐rhod‐1 was taken every second to illustrate the relative concentration of Ca^2+^. To test the mechanical sensitivity of the mMscL channel, various levels of loads including 0.5, 2, and 5 N were applied to the cultured cells, respectively (Movie S1, Supporting Information). It means that each cell was exposed to pressures of 100, 400, and 1000 KPa, accordingly. Upon a 2 N load stimulation, the mMscL‐dependent Ca^2+^ influx was clearly detected in the target cell. Compared to the 2 N load, the 5 N load did not significantly increase the activation of the mMscL channel. Additionally, the Ca^2+^ influx was affected by mechanical loading, but not by the frequency or duration of such loading (Figure 2D,E). Since the activation of calcium channel mMscL by mechanical force is transient,^[^ ^14^ ^a]^ once the peak concentration was reached, there was a reduction in Ca^2+^ concentration. However, the weight‐pressing induced response was abrogated when mMscL‐expressing cells were imaged in Ca^2+^‐free culture (Figure 2D,E). Moreover, both force from flow cytometry and centrifugal demonstrated that applying 400 KPa pressure effectively induced Ca^2+^ influx in mMscL expressing cells (Figure S2, Supporting Information). These results demonstrated that 2 N loads on the 3D cell petri dish, which is ≈400 KPa pressure per cell was suitable to activate the Ca^2+^ influx switch. Therefore, a pressure of 400 KPa per cell was used for mechanical stimulation in subsequent experiments.

### 2.2 Characterization of the Mechanogenetic Circuit for Programmed Tumor Cell Apoptosis

**Figure 3:**
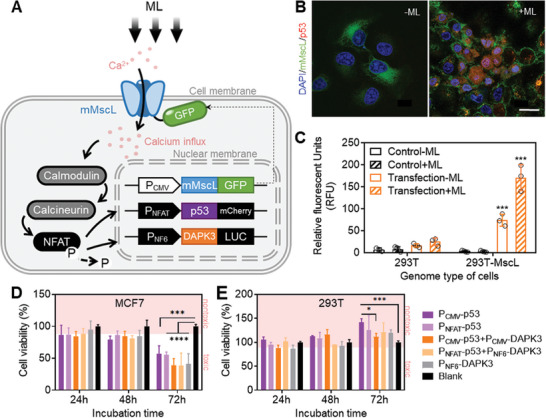
Design and characterization of the mechanogenetic circuit in mammalian cells. A) Schematic drawing of ML‐induced transcriptional activation and gene expression. The mechanical pressure stimulated Ca^2+^ influx and thus triggered the downstream pathways, including calmodulin/calcineurin activation, NFAT dephosphorylation, and translocation into the nucleus. The wild type NFAT promoter (P_NFAT_) and enhanced NFAT promoter (P_NF6_) could response to concentration fluctuations of Ca^2+^ and initiated the expression of 3′‐end tumor suppressors of p53 and DAPK3, respectively. B) Representative fluorescence microscopy images showing mechanical loading switch of the mechanogenetic circuit. The NFAT downstream gene p53 (P_NFAT_ fused at 5′ end and mCherry as the fluorescent reporter fused at 3′ end) expressed only with ML stimulation. The ML was exerted via centrifugation for 5 min  h^−1^ and repeating for 12 times. Scale bar: 20 µm. C) The bar graphs that represent the fluorescence intensity of LUC and DAPK3 expression level in ML‐induced mMscL expressing cells (n = 3). (D‐E) Cell counting kit‐8 (CCK8) assay to examine the effect of the mechanogenetic circuit. The pink area represented where the cell viability was >90%. The blank group represented the cells expressing mMscL stably but without introducing additional downstream targets of NFAT. Error bars indicate SEM, ^*^ *p* < 0.05, ^***^ *p* < 0.001, ^****^ *p* < 0.0001, and from two‐tailed student t test.

To characterize the effect of the ML‐induced Ca^2+^ influx on genetic activities, one stably expressed transcription regulator NFAT was adopted.^[^ ^17^ ^]^ The transcriptional activity of NFAT can be initiated by the Ca^2+^‐mediated dephosphorylation.^[^ ^18^ ^]^ Two reported NFAT‐binding promoters were used here, in which the primitive NFAT promoter (P_NFAT_) has one NFAT binding site, while the enhanced NFAT promoter (P_NF6_) contains six consecutive NFAT binding sites. Therefore, the relative transcriptional strength of the promoters was compared as P_CMV_:P_NF6_:P_NFAT_, with a ratio of ≈6:6:1 (Ref.[^[^ ^19^ ^]^]). Two vital effectors, p53, and DAPK3, were chosen as downstream targets for Ca^2+^ influx activation. These two proteins play a critical role in monitoring carcinogenesis and inhibiting cancer development, while they are usually mutated in cancer cells. As shown in **Figure** **3**A, P_NFAT_ was used to transcribe the expression of p53 gene (P_NFAT_‐p53). Since p53 is nuclear localized and its overexpression is responsible for various cellular metabolic disorders, the promoter with weaker transcriptional strength is suitable for activating p53 expression.^[^ ^20^ ^]^ As for DAPK3, it can localize to either the nucleus to synergize with the pro‐apoptotic effects of p53, or to the cytoplasm. It can even be exocytosed to the outside of the cell, transmitting signs of cancer in the extracellular matrix to induce cellular autophagy, and immune recognition. Therefore, the stronger P_NF6_ promoter was chosen to initiate DAPK3 expression (P_NF6_‐DAPK3).^[^ ^21^ ^]^ To quantitatively detect the protein expression levels, the mCherry gene and the firefly luciferase (LUC) gene were cloned to the 3′‐end of p53 and DAPK3 genes, respectively.

Next, the expression plasmids of p53 and DAPK3 were stably transfected into mMscL‐GFP expression cells, including 293T and MCF7 cell lines. The application of ML significantly activated P_NFAT_ and initiated p53 expression in mMscL‐expressing cells, as indicated by the fluorescence signals localized on the cell membrane and nucleus (Figure 3B; Figure S3, Supporting Information). These results indicated that the mMscL channel and inducible promoter P_NFAT_ can be integrated, which synergized with the endogenous molecular network. This integration allowed for the sensing of ML stimulate, thereby directing the gene expression. The transcription of P_NF6_ and thus expression of DAPK3 were also induced by mechanical stimulation, as evidenced by the intensity of bioluminescence produced by LUC (Figure 3C). However, in mMscL‐expressing 293T cells, the target protein DAPK3‐LUC was expressed even without mechanical force stimulation, perhaps due to the high binding affinity of NFAT to P_NF6_. Therefore, it is reasonably speculated that a small amount of calcium ions can trigger DAPK3 expression in the presence of mMscL. Collectively, these results indicated that the ML‐induced Ca^2+^ influx can initiate strong expression of engineered target genes and induce robust mechanogenetic effects.

Then, MCF7 and 293T cells stably expressing mMscL proteins were transfected with P_CMV_‐P53, P_NFAT_‐P53, P_CMV_‐DAPK3, or P_NF6_‐DAPK3. As shown in Figure 3A, six groups of mechanogenetic circuits were established and tested to determine the effect of mechanical stimulation toward cell viability. The cells were subjected to ML stimulation for 5 min h^−1^ over a period of 12 h, followed by incubation for 24, 48, and 72 h, respectively. As shown in Figure 3D,E, the viability of 293T cells upon activation of p53 and DAPK3 expression remained higher than 90%, and the viability of cells expressing p53 even increased by 1.2‐ to 1.5 fold after incubation for 72 h. The results indicated that the activation of the mechanogenetic circuits cannot impede growth of normal mammalian cell lines. However, as for the MCF7 cell lines, the viability of p53 and DAPK3‐expressing cells decreased >2 fold, when compared with controls without p53 and DAPK3 expression cassettes. Additionally, the cytotoxic potency of antitumor proteins induced by P_NFAT_ was equivalent to that of the constitutive P_CMV_ overexpression in MCF7 cells. In the 293T cells, the 72 h‐ cell viability of P_CMV_‐p53 group was significantly increased, which was different from other groups. This indicated that ML‐mediated P_NFAT_‐induced expression is more moderate and controllable than constitutive expression, reflecting the reversible manipulation ability of mechanogenetic circuits. Besides, there was no significant distinction between the outcomes of only p53 expression and the co‐expression of p53 and DAPK3. These findings suggested that ML‐induced calcium influx might be saturated for NFAT dephosphorylation. So it cannot increase the expression level of tumor cell killer proteins by increasing the downstream gene expression cassettes. After 12 h ML stimulation, MCF7 cell viability was significantly decreased to 39% of the blank group, clearly indicating that the expression of antitumor protein may lead to a rapid, and effective cell‐apoptosis effect mediated by ML stimulation.

Furthermore, we investigated the tumor cell apoptosis effects of mechanogenetic circuit expressing cells under the control of ML. We simultaneously transfected the P_NFAT_‐p53 and P_NF6_‐DAPK3 cassettes into MCF7 cells and 293T cells, which stably express the mMscL protein. After 12 h of stimulation and 12 h of incubation, the transcript levels of p53 and its downstream apoptosis‐related proteins were examined (Figure S4A–H, Supporting Information). Under the control of mechanogenetic circuit, the expression strength of p53 induced by ML was ≈1.5 fold higher than in unstimulated cells, regardless of the cell type. To evaluate the impact of mechanogenetic circuits, the transcription levels of downstream apoptosis‐related proteins were detected, such as p53 downstream cell cycle regulatory protein (p21), p53 upregulated modulator of apoptosis (PUMA), and the TNF‐related apoptosis‐inducing ligand receptor (killer). Significantly, the expression of apoptosis‐related proteins remarkably increased in MCF7 tumor cells, whereas no such change was observed in normal 293T cells. The results demonstrated that the synthesis of p53 stimulated by ML is more effective in activating its downstream pathways in tumor cells. As reported, the MCF7 cells only possess the mutant p53, which differs from normal cells.^[^ ^22^ ^]^ The overexpression of ML‐stimulated p53 directly dominated the metabolism of cancer cells and significantly activated the apoptotic pathway, thereby effectively killing the tumor cells. In addition, protein immunoblotting experiments similarly validated these findings at the protein level (Figure S4I–O, Supporting Information). By validating the marker proteins of the Caspase‐dependent mitochondrial apoptotic pathway, it was found that the expression of the apoptotic precursor, Caspase9, was not regulated. However, the expression level of the cleavage product of Caspase3, the downstream protein of Caspase9, was slightly increased. It suggested that the function of Caspase9 shearing Caspase3 was affected by the elevation of p53 expression, which in turn activated the programmed apoptosis of tumor cells. The resulting insignificant changes in the expression of immune‐related marker protein (STING) might be attributed to the single cell type and lack of signal exchange between multiple cells. We further tested the mouse breast tumor cell 4T1 for expression of p53 and downstream apoptosis‐related proteins (Figure S5, Supporting Information), confirming that the ML stimulated mechanical genetic circuit is efficient to activate the tumor therapy function. Although the half‐life of p53 was reported to be <20 min,^[^ ^23^ ^]^ the half‐life of ML stimulated p53 was over 3 h (Figure S5, Supporting Information). In both MCF7 and 4T1 cells, the maintaining time of apoptosis‐related protein exceeded that of p53. These results indicated that the mechanical genetic circuits can ensure the sustained therapeutic effect and offer the reversible regulatory function for tumor cells. Moreover, this approach has broad applicability for cancer therapy and is compatible with various species.

### 2.3 Implementing of the Mechanogenetic Circuit for In Vivo Breast Tumor Treatment

**Figure 4:**
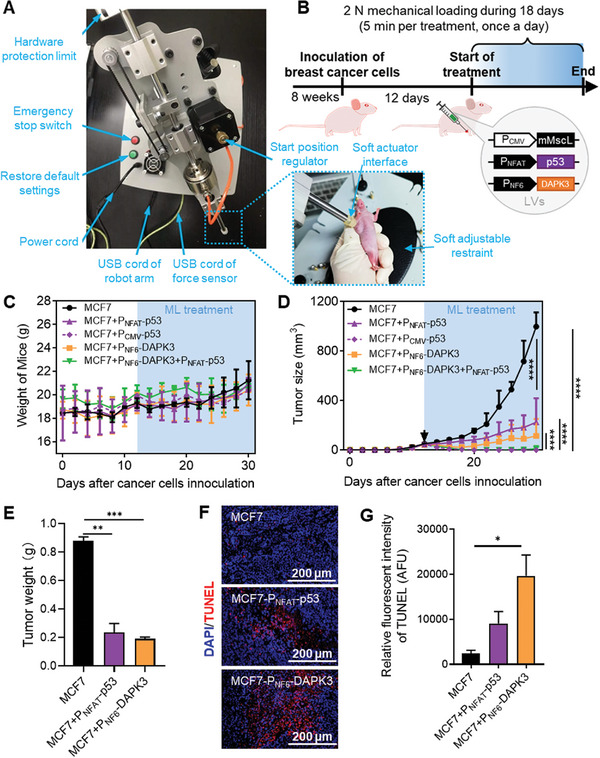
Treatment of tumor‐bearing Balb/c‐nude mice via cyclic ML stimulation coupled with mechanosensitive p53 and DAPK3 signaling pathway. A) Photograph of robot arm for mechanical loading on mice models. The robotic arm equipped with a soft‐interface actuator, a force sensor, and a microcontroller regulated by computer. The demonstration of the actuator positioned toward the tumor tissue. The right picture shows the mechanical loading process for cancer treatment. B) Schematic diagram of the experimental setup. The breast tumor cells MCF7 were inoculated to 8 week‐old mouse. The treatment by transfected mechanogenetic circuit and giving ML was started when the tumor volume reached up to 50 mm^3^. C–E) Quantification of mouse weight, tumor volume, and tumor weight (n = 6). F) Immunofluorescence of tumor tissue sections. The tumor tissue was separated from mouse on day 34 and detected by TdT‐mediated dUTP‐biotin nick end labeling (TUNEL) staining. G) Quantification of TUNEL fluorescence intensity. Error bars indicate SEM, ^*^ *p*<0.05, ^**^ *p* <0.01, ^***^ *p* <0.001, ^****^ *p* <0.0001, and from two‐tailed student t‐test.

After confirming the expected efficacy of the mechanogenetic circuit in tumor killing in vitro, we proceeded to evaluate its in vivo biological function for cancer treatment. We developed a system for exact control of the ML to ensure that a stable and uniform mechanical force was administrated to tumor‐bearing mice (**Figure** **4**A). The system uses a robotic arm that is controlled by feedback signals from a force sensor (Movie S2, Supporting Information). As for the Balb/c mice bearing 4T1 tumor cells, one group of mice containing the mechanogenetic circuit was treated with 2 N mechanical loading once a day, and another group was treated with chemical drug gemcitabine daily. In comparison, both groups showed similar treatment efficacy at the end of treatment (Figure S6A–C, Supporting Information), as indicated by the tumor sizes and staining of tumor tissues. Besides, the combined treatment with both gemcitabine and mMscL‐mediated mechanogenetic circuit showed the best therapeutic effect (Figure S6D–F, Supporting Information), suggesting the excellent compatibility of this mechanogenetic circuit with other chemotherapeutics. To further verify the cancer treatment effect of ML stimulation, the mice models bearing subcutaneous human breast cancer cells MCF7 were established (Figure S7, Supporting Information). In the mice loaded with 4T1 or MCF7 breast cancer, both the target gene *p53* and the tumor apoptosis‐associated gene *killer* were mechanically activated (Figures S6E and S7D, Supporting Information). However, the expression strength of the inducible promoter P_NFAT_ was lower than that of the constitutive promoter P_CMV_ (Figure S7D, Supporting Information). Thus, the ML‐activated p53 expression significantly slowed tumor growth and increased the lifespan of mice in 1.7 fold, although the treatment effect was slightly lower than the stably expressed p53 (P_CMV_‐p53). It might be caused by the intermittent activation of the mechanogenetic circuit, instead of the continuous activation. Due to the reversible regulatory function of mechanogenetics, the inducible expression of p53 may not be adequate for completely eliminating cancer cells.

Furthermore, in order to enhance the cancer treatment effect of the mechanogenetic circuit, we explored to manipulate cooperative gene expression in vivo. 8 week‐old Balb/c‐nude mice were used and each mouse with weight of 20 g was injected with 1 × 10^6^ MCF7 cells. Once the tumor reached a volume of 50 mm^3^, the mechanogenetic circuits containing tumor inhibition effectors of P_NAFT_‐p53 and P_NF6_‐DAPK3 were transfected into subcutaneous breast tumor tissues via lentiviral vectors (LVs) (Figure 4B). The robotic‐actuation treatment was then applied on the day following injection of LVs, with a reciprocating mechanical force of 2 N and a duration time of 5 min d^−1^. A significant disparity in the sizes of axillary‐loaded tumors was observed in 18 days after treatment (Figure 4C,D). Compared to the group of mice without LVs transfection (denoted as MCF7 group), the tumor growth was obviously inhibited in all groups with LVs transfection (Figure S8, Supporting Information). Among them, the group that constitutively expressed p53 (MCF7+P_CMV_‐p53) and the group with two induced anti‐tumor proteins (MCF7+P_NF6_‐DAPK3+P_NFAT_‐p53) hardly showed the existence of tumor. This result suggested that DAPK3 can assist p53 and inhibit tumor growth, and there is great potential for NFAT to activate P_NF6_ and P_NFAT_ at the same time. The apoptosis fluorescence and tumor weights demonstrated that the antitumor proteins p53 or DAPK3 can independently slow tumor growth (Figure 4E–G). Specifically, the isolated expression of DAPK3 exhibited superior therapeutic efficacy compared to p53, potentially due to the higher transcription strength of P_NF6_. The results demonstrated that the synergetic expression of downstream effectors can effectively enhance the regulatory strength of mechanogenetic and inhibit tumor growth.

### 2.4 Customized Design of the Mechanogenetic Therapeutic Strategy for Tumors at Different Stages

**Figure 5:**
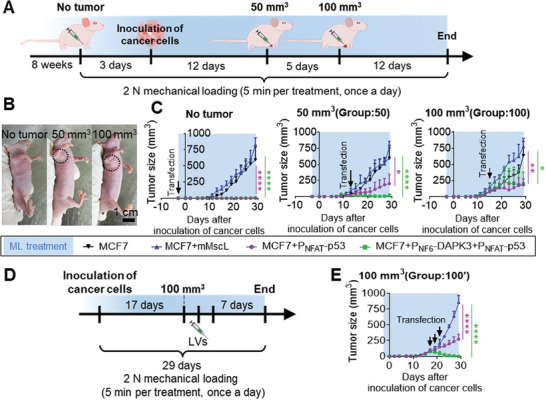
The enhancement of mechanogenetic therapy for tumors at different stages. A) Experimental design and time line of treatment of tumors at different stages via mechanical loading and LVs transfection. The transfection of LVs was administrated at three stages, including 3 days (No tumor) before inoculating tumor cells to the mice, as well the 12 days (tumor size reached to 50 mm^3^) and 17 days (tumor size reached to 100 mm^3^) after the inoculation of tumor cells. To exclude confounding factors, all subgroups of mice were treated by ML from 3 days before tumor cells inoculation. B) Photographs were taken of the tumor‐bearing mice when the LVs transfection started. Tumor tissues were marked in black dotted circles. C) The tumor volume of mice from three treatment groups (n = 5). The black arrow indicated the time point of LVs transfection. D) Schematic diagram of the customized treatment for tumors, whose volume were up to 100 mm^3^. The experiment group was named as “ 100′ ”. The LVs injection was administrated at a therapeutic interval of 1 day from day 17 to 23. E) The tumor growth of mice in “ 100′ ” (n = 5). Error bars indicate SEM, ^*^ *p *< 0.05, ^**^ *p* < 0.01, ^****^ *p* < 0.0001, and from two‐tailed student t‐test.

To further verify the feasibility of the mechanogenetic therapy for treating tumors at different stages, we examined the impact of the initiation timing of anti‐tumor protein expression in treatment. We utilized the MCF7 breast cell line to construct the tumor bearing Balb/c‐nude mice (**Figure** **5A,B**). Once the tumors reached an average size of ≈50, 100, and 500 mm^3^, we performed LVs injection with mechanogenetic circuits for one time. One experimental group was transfected the mechanogenetic circuits before tumor inoculation. We found that tumor tissue treated with ML to activate the antitumor expressing pathway significantly prevented cancer development (Figure 5C left). Particularly, when tumor volume was <50 mm^3^, the simultaneous induction of p53 and DAPK3 expression (MCF7+P_NF6_‐DAPK3+P_NFAT_‐p53) resulted in almost complete tumor eradication (Figure 5C middle). When the tumor developed to a volume up to 100 mm^3^, there was a substantial inhibitory impact on tumor growth within 1 week after mechanical initiation of the anti‐tumor pathway. However, the tumor tissues resumed their original growth rate afterward (Figure 5C right). Immunofluorescence staining of tumor tissues revealed that the transfection efficiency of LVs might be limited by one‐time injection (Figure S9, Supporting Information). This similar phenomenon was also observed when the one‐time injection strategy was applied to large‐volume tumors with size over 500 mm^3^ (Figure S10A,B, Supporting Information). Moreover, the therapeutic efficacy of inducible anti‐tumor proteins (P_NFAT_‐p53+P_NF6_‐DAPK3) was comparable to that of the constitutive group (P_CMV_‐p53), indicating that the timing of LVs injection did not affect the therapeutic efficacy (Figure S11, Supporting Information). It also proved that the transfection ability of LVs might be insufficient for large tumor therapy.

To address these limitations, the mechanogenetic antitumor circuit was applied with increased transfection times of LVs (Figure 5D; Figure S10A, Supporting Information). When tumors volume reached to 100 mm^3^ (referred as 100′) or 500 mm^3^ (referred as 500′), the injections of LVs were administered three times interval over 1 day along with subsequent mechanical stimulations. The tumor tissue up to 100 mm^3^ was completely eliminated within 1 week by inducing the expression of both p53 and DAPK3 (Figure 5E). Furthermore, the growth rate of the 100 mm^3^ tumors was significantly slowed down through transfection of mMscL and p53 expression cassette. In addition, for the tumor up to 500 mm^3^, the ML‐induced p53, and DAPK3 significantly slowed the tumor growth under the increase of LVs transfection and prolonged the terminal life (Figure S10B,C, Supporting Information). Immunofluorescence staining of tumor tissues revealed that the continuous injection of LVs could transfect the mechanogenetic circuit more efficiently in tumor section (Figure S12, Supporting Information). In addition, the expression of apoptosis‐related genes significantly increased in tumor tissue as documented by the immunoblotting and flow cytometry (Figure S13, Supporting Information). HE staining detected that there was no negative impacts on cardiac and pulmonary function, as well as hepatosplenomegaly caused by tumor overgrowth (Figure S14, Supporting Information). It showed that the consecutive transfections of mechanogenetic circuit were safe and could not interfere with the normal function of main organs. Therefore, the mechanogenetic therapy can significantly slow the growth of tumor on demand and effectively addresses the limitations of conventional gene therapy, particularly in inhibiting the growth of larger tumors.

## 3 Conclusion

We here employed a novel non‐invasive therapeutic approach utilizing mechanical stimulation mediated by robotic actuation for low‐energy‐consuming physical therapy. Initially, a cell imaging system employing 3D cell culture and confocal microscopy for observing the conversion from mechanical signals into biochemical signals was established. From the system, the stimulation of robotic actuation was quantified and proved to activate cell activities. Subsequently, a mechanogenetic circuit was engineered in mammalian cells to establish a direct link between mechanical signals and disease treatment. The circuit included the mechanosensitive channel mMscL, the transcriptional factor NFAT, and downstream therapeutic genes initiated by P_NFAT_ or P_NF6_. Upon transfecting this mechanogenetic circuit into mice tissues and stimulating by robotic actuation, target proteins were expressed for therapeutic purposes. Our study demonstrated that this mechanogenetic circuit could efficiently transmit and utilize mild mechanical signals via robotic actuation, without the need of high‐power devices and adjuvant medication. Moreover, the robotic actuation‐mediated mechanogenetic therapy holds great potential for expanding physical therapy techniques and presenting pioneering approaches for gene therapy.

The anti‐tumor proteins p53 and DAPK3 were transfected into mammalian cells through LVs, serving as the therapeutic genes of the mechanogenetic circuit. The two anti‐tumor protein system exhibited a synergistic effect, enhancing the regulatory effects of p53 pathways and activating the mitochondria‐mediated apoptotic pathway in tumor cells. The results demonstrated that activation of the mechanogenetic circuit efficiently inhibited tumor growth while promoting normal cell growth, without any induction of inflammatory reactions in transfected tissues. This strategy specifically targeted large‐volume tumors that are difficult to cure using standard gene therapy. Remarkably, the MCF7 tumor (volume ≤ 100 mm^3^) of Balb/c‐nude mice can be totally cured by transfecting mechanogenetics circuit and treating daily with quantitative robotic actuation in a form of 5 min treatment over the course of 14 days. Furthermore, larger tumors (volume = 500 mm^3^) could be effectively suppressed. In stark contrast to conventional gene therapy techniques, this robotic actuation‐mediated mechanogenetic strategy is more effective in regulating gene expression and treating large tumors. This offers a secure and on‐demand approach to cancer treatment.

By the established robotic actuation device and mechanogenetic circuit transfection module, this work presents a practical and promising therapeutic approach for cancer treatment. Robotic actuation is a gentle and natural mechanical force that is convenient and compatible for patients to accept. In addition, the mechanogenetic process mediated by robotic actuation achieves a higher energy conversion efficiency to achieve therapeutic goals. Compared to existing genetic techniques such as sonogenetics, optogenetics, and electrogenetics, robotic‐actuation mediated mechanogenetics avoids the damage caused by energy accumulation. In the future, the intricate relationship between mechanical and biochemical signals will be investigated systematically. The therapeutic effect of mechanogenetic therapy could be further strengthed by improving gene transcription efficiency, designing diverse therapeutic effector genes, prolonging the lifespan of therapeutic proteins, or combining with other physical therapies.^[^ ^24^ ^]^ Furthermore, this advancement might be helpful to reveal new therapeutic strategies for addressing challenges in mechanical signal transduction‐related diseases.

## Conflict of Interest

The authors declare no conflict of interest

## Supporting information

Supporting Information

Supplemental Movie 1

Supplemental Movie 2

## Data Availability

The data that support the findings of this study are available in the supplementary material of this article.
