# Supplementary material for: Robotic Actuation‐Mediated Quantitative Mechanogenetics for Noninvasive and On‐Demand Cancer Therapy
Source: Adv Sci (Weinh). 2024 Mar 21;11(23):2401611. doi: 10.1002/advs.202401611 (PMC11186056; doi:10.1002/advs.202401611)
Supplement: Supplementary file 1 — Supporting Information [file ADVS-11-2401611-s002.pdf]

ADVANCED SCIENCE
Open Access

Supporting Information

for *Adv. Sci.*, DOI 10.1002/advs.202401611

Robotic Actuation-Mediated Quantitative Mechanogenetics for Noninvasive and On-Demand Cancer Therapy

Yangyi Liu, Jingjing Li, Yi Zhang, Fan Wang, Juanjuan Su*, Chao Ma, Shuyi Zhang, Yanan Du, Chunhai Fan, Hongjie Zhang and Kai Liu**

Supporting Information

Robotic Actuation-mediated Quantitative Mechanogenetics for Noninvasive and On-demand Cancer Therapy

Table of Contents

Experimental Information 2
Materials 2
Methods 2
Supplementary Figures, Tables and Movies 5
Figure S1 5
Figure S2 6
Figure S3 7
Figure S4 8
Figure S5 9
Figure S6 10
Figure S7 12
Figure S8 13
Figure S9 14
Figure S10 15
Figure S11 17
Figure S12 17
Figure S13 18
Figure S14 19
Table S1 20
Table S2 22
Table S3 23
Movie S1 legend 23
Movie S2 legend 233
References 23

Experimental Information

1. Materials

a) Cell lines and experimental animals

293T, 4T1 and MCF7 cell lines were purchased from the American Type Culture Collection (ATCC). The identities of the cell lines were verified by short tandem repeat analysis. All cell lines were confirmed to be mycoplasma free. Female Balb/c-nude mice were bought from Beijing Vitalstar Biotechnology Co., Ltd.

b) Gene and lentiviral vectors

The sequence information can be found in the Protein Data Bank (Supplementary Table1). The encoding sequences of mMscL, p53 and DAPK3, the promoter sequence of P_{CMV}, P_{NFAT} and P_{NF6}, and the lentiviral vectors (LVs) containing the resistance genes of Geneticin and Puromycin were also generated by Geneviewz. The GFP, mCherry, and luciferase were amplified from plasmids maintained in the laboratory. All the primers utilized in this study were obtained from Tsingke (refer to Supplementary Table2).

c) Reagents

All reagents and solvents were purchased from commercial sources and used as received without other purification unless otherwise noted. NaCl, KCl, $MgCl_2 \cdot 6H_2O$, $CaCl_2$ and Glucose were bought from Sinopharm Chemical Reagent Co., Ltd. Methanol were purchased from Shanghai Titan Scientific Co., Ltd. 30% PAGE Pre-solution and 4% paraformaldehyde were bought from Beijing Solarbio Science & Technology Co., Ltd. DMEM, Earle's balanced salt solution (EBSS), Phosphate-buffered saline (PBS), Trypsin and 100× penicillin/streptomycin mixture were bought from Biological Industries (BIOIND) Ltd. Fetal bovine serum (FBS) were bought from NEWZERUM Ltd. N-2-hydroxyethylpiperazine-N-ethane-sulphonicacid (HEPES), fluorescent calcium indicator X-rhod-1 and 4′,6-diamidino-2-phenylindole (DAPI) were purchased from Thermo Fisher Scientific Inc. RT-PCR kit, Universal RNA Extraction Kit and SYBR Green PCR kit were purchased from Takara Biotechnology Co., Ltd. Transfection reagent polyetherimide (PEI) [1] was bought from Sigma-Aldrich Company Ltd. ECL western blotting substrate reagent and PVDF blotting membrane were purchased from Amersham Biosciences, Inc. Basement Membrane Matrix (3D-culture media) was bought from Corning Inc. Primary antibodies and HRP-conjugated anti-rabbit secondary antibodies were purchased from Bioss Inc. (Supplementary Table3) The Alexa Fluor® 488 and Alexa Fluor® 647 kit for preparing direct fluorescent antibodies were purchased from Abcam Inc. Mechanical loading equipment was constructed in this work, in which pressure sensor was purchased from CHINO SENSOR CO., LTD.

2. Methods

a) Cell culture condition and transfection

Cells were maintained in DMEM with 10% fetal bovine serum and 1% penicillin/streptomycin mixture at 37 ℃, 5% CO_2. For cell transfection, 2×10^5 cells were plated in a 12-well plate and cultured for 24 hours. The plasmids were then transfected into the cells using a 25-kDa branched transfection reagent, and the cells were then cultured for 48 hours to express the target proteins.

b) The inoculation of tumor cells and lentivirus infection

Animal experiments were performed on 8-week-old female Balb/c nude mice according to the statutory requirements of the People's Republic of China (GB14925−2010). The mice were subcutaneously inoculated with 100 μL of tumor cell suspension in the right upper limb. The number of cells was about 2×10^6. The mice were divided into four groups with different

treatments. There were five to six mice in each group. All animal experiments were conducted in compliance with the Animal Management Rules of the Ministry of Health of the People's Republic of China and with the approval of the Institutional Animal Care and Use Committee of the Animal Experiment Center of Tsinghua University. The assigned approval/accreditation number of the laboratory is THU-02-2023-0160A.

c) RNA extraction and RT-PCR

Total RNA was extracted using the Universal RNA Extraction Kit. 1μg of RNA was used for cDNA synthesis using the RT-PCR kit according to the manufacturer's instructions, followed by PCR amplification using a SYBR Green PCR kit and designed primers (the sequences are provided in Supplementary Table2). The amplification program was consisted of incubations at 95 ℃ for 15 seconds and then 60 ℃ for 60 seconds. GAPDH was used as an endogenous control. All cycle threshold (CT) values were determined by real time using CFX96™ Real-Time PCR Detection (Bio-Rad, CA).

d) Western blot analysis

Proteins were separated from cells or tissue lysates, and the mixed proteins were then analyzed by 15% SDS-PAGE and transferred on the 0.2 μm PVDF film. The membrane was then washed briefly with methanol and dried for 15 min to enhance the protein binding. After the drying step, the membranes were reactivated with methanol. Nonspecific binding sites were then blocked by immersing the membrane in TBS-T buffer containing 5 % nonfat dry milk. The membrane was washed briefly and incubated with the primary antibody (Supplementary Tab. 3) in TBS-T buffer for 1 hour. The membrane was then rinsed three times for 5 min each time with TBS-T buffer, and incubated with TBS-T buffer containing HRP-conjugated anti-rabbit secondary antibody (1:5,000 dilution) for 1 hour. Subsequently, the membrane was washed three times for 5 min each time with TBS-T buffer, and incubated with ECL western blotting substrate reagent. Finally, it was exposed in a dark room for 1 min and the signal is collected on the chemiluminescence camera ChemiScope61004 (Clinx, CN).

e) Fluorescence microscopy of proteins

GFP/mCherry expressing cells were grown on coverslips, washed with PBS and treated with EBSS for 4 hours. Cells were then rinsed three times with PBS and fixed with 4% paraformaldehyde for 20 min at room temperature. Images were acquired with an A1R HD25 (Nikon, JPN) confocal laser scanning microscope that had Ar-488, 561, and DAPI laser excitation capabilities.

f) Fluorescence imaging of intracellular Ca^{2+}

Cells were incubated with 4 mM of the fluorescent calcium indicator X-rhod-1 (Product code: X-14210) for 30 min at 37 ℃. The fluorescence imaging instrument was equipped with a force-stimulation component, enabling detection of the changes in intracellular calcium ion concentration by the alteration in the fluorescence signal at 561 nm. During the imaging of Ca^{2+}, the cells were placed in a buffer solution with 130 mM NaCl, 2 mM $MgCl_2$, 4.5 mM KCl, 2 mM $CaCl_2$, 10 mM Glucose, and 20 mM HEPES, (pH 7.4).

g) Staining and flow-cytometry

Section staining was performed by Servicebio Company. Cells and tumor tissues were collected from ML stimulation and transfection experiment and homogenized into single-cell suspensions according to the established protocol [2]. Subsequently, the cell suspensions were stained with different antibodies according to the standard protocol. All of the flow cytometry

analyses were conducted using the CytoFLEX LX Flow Cytometer (Beckman, US) and were performed using FlowJo 10.

h) In vitro mechanical loading

ML stimulation by weights. For Fig. 1d-e, cells were seeded on a glass bottom of 35 mm petri dish. For Supplementary Movie1, cells were suspended in 1 mL 3D-culture media at 4 ℃ and gelled at 37 ℃. After 24 hours of culture, all Petri dishes were washed twice with PBS. The calcium indicator and calcium imaging buffer were added to the dishes before the mechanical loading. After opening the dish, a piece of cell glass slide was lightly covered on the cells. The weights were put down and lifted with elbow tweezers.

ML stimulation by centrifugation. Cells were seeded in a 12-well plate and cultured with 1 mL DMEM + 10% FBS media. The plate was placed in the centrifugal basket and then centrifuged at 150 ×g for 5 min/hour at 37 ℃, and this step was repeated 12 times.

ML stimulation by flow cytometry. Cells were incubated with 4 mM Ca^{2+} imaging indicator for 30 min at 37 ℃, and then resuspended by trypsin. Subsequently, the resuspended cells were filtered using a 400-mesh sieve and dispersed into 1.5 ml EP tubes with PBS, and the fluorescence intensity at 561 nm was detected at flow rates of 10, 60, and 120 μL/min.

i) Mechanical loading treatment of mice

The treatment was performed every day. Each mouse was captured an overturned position to expose the axilla of the left upper limb. A robotic arm was used to apply a mechanical force to the tumor tissue in a back and forth way. The applied mechanical loading was 2 N, with the robotic arm lifting to a height of 1 cm at a speed of 1 cm/s. The pressing treatment was employed for 5 min per day, with a pressing frequency of 20 times per min. If both lentiviruses transfection and mechanical loading was to be applied on the same day, the mechanical loading would be performed first.

j) Statistical analysis

Statistical significance between two groups was determined with two-tailed Student's t-test. All statistical analyses were performed using the GraphPad Prism 8 software. Data are presented as the mean ± SEM. Generally, all experiments were carried out with n≥ 3 biological replicates. P values of <0.05 was considered statistically significant.

Supplementary Figures, Tables and Movies

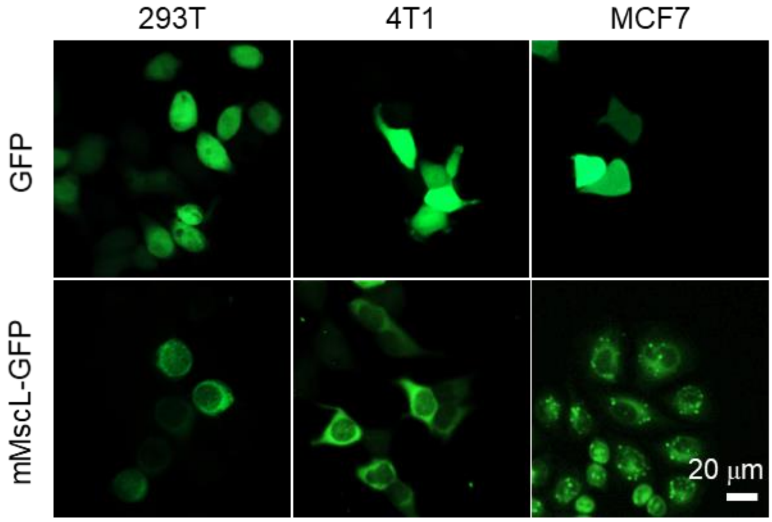

Figure S1. Expression level and localization of mMscL in mammalian cells.

Fluorescence microscopy image of cells after transfection and culture for 48 h. The cells were fixed, and the localization of the GFP-fused mMscL was visualized. Cells transfected with the GFP coding sequence served as a control. Scale bar: 20 μm.

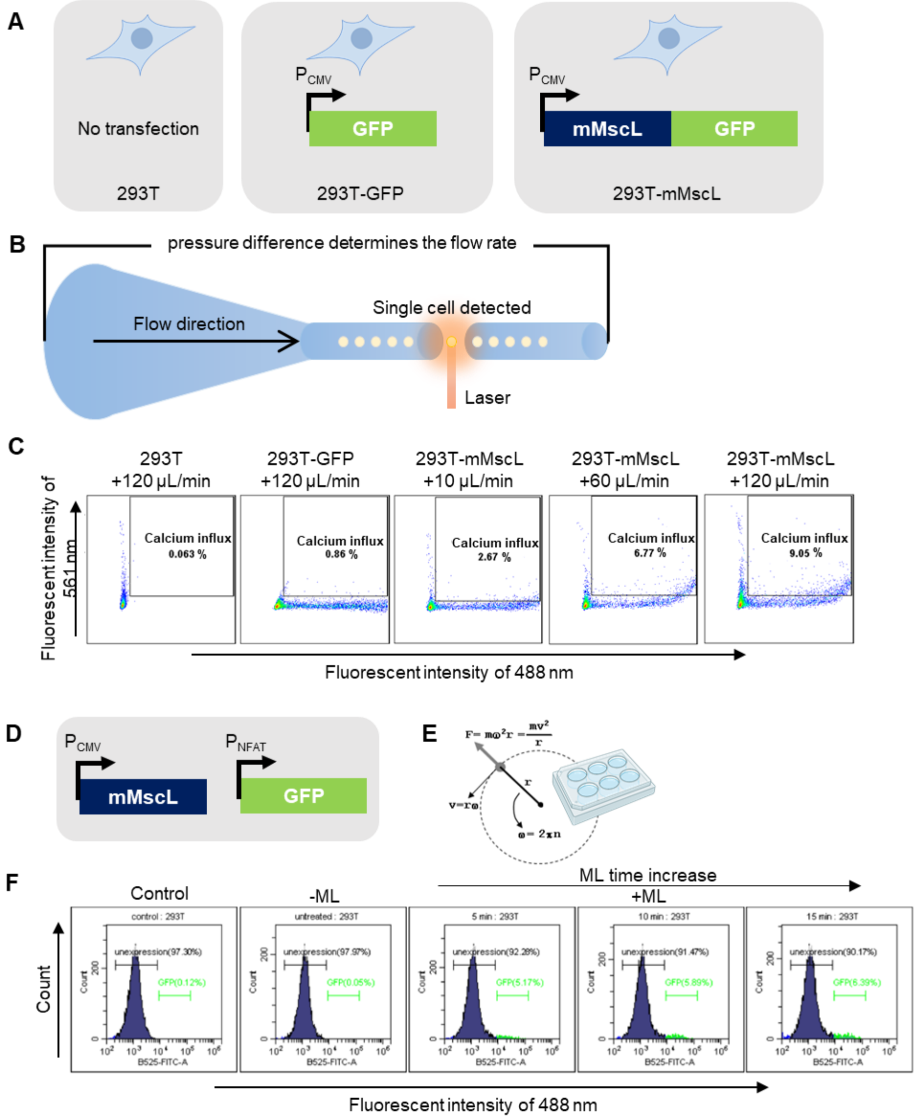

Figure S2. Assessment of the sensitivity of mMscL. (A-C) Ca^{2+} influx of mMscL-GFP-expressing 293T cells. The 293T cells without transfection was named as “293T”. The GFP expressing 293T cell was named as “293T-GFP”. The mMscL-GFP expressing 293T cell was named as “293T-mMscL”. All cells were cultured with X-rhod-1 for 12 h, washed twice with PBS, and then analyzed for 561 nm fluorescent signals using flow cytometry. The 293T cells that were transfected P_{CMV}-GFP did not show any significant changes in calcium ion concentration at flow rates of 120 µL/min. However, the 293T-mMscL cells showed significant changes in intracellular calcium flow, with increases in calcium ion concentration at flow rates of 10 µL/min, 60 µL/min or 120 µL/min of up to 3.1-fold, 7.9-fold and 10.5-fold, respectively, compared to the 293T-GFP cells. (D-E) Effect of ML stimulation duration on GFP reporter expression. The 293T cells transfected with mMscL and the gene activation cassette P_{NFAT}-GFP was detected by flow cytometry. The cells were stimulated by centrifugation for 5, 10, or 15 min per hour and were then detected after 12 hours of static culture.

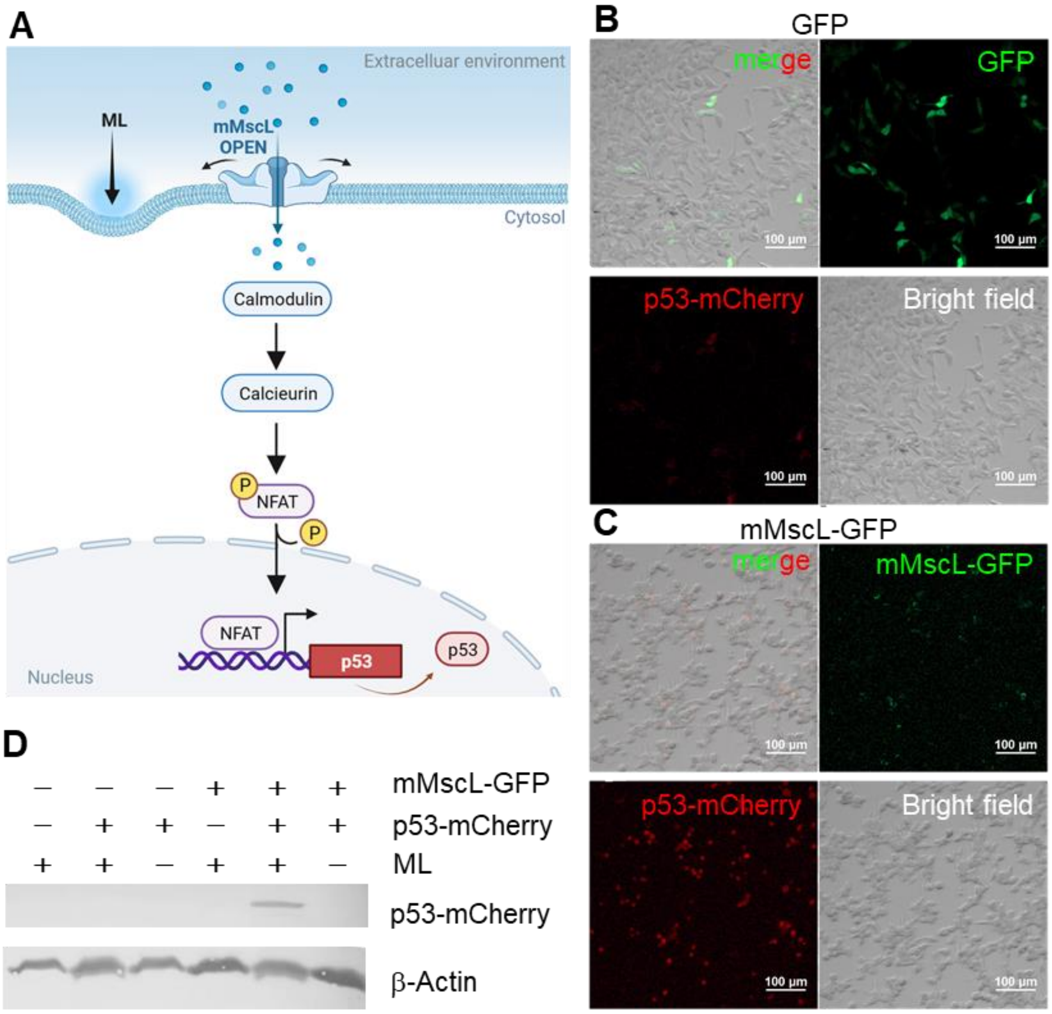

Figure S3 Expression of downstream genes stimulated by mechanogenetic switching in 293T cells. (A) A schematic diagram of the process used to establish a mechanically sensitive gene expression pathway in cells stably expressing mMscL. This figure was created with reference to pictures in BioRender.com. (B-C) Fluorescence microscopy imaging of cells transfected with the GFP or mMscL-GFP coding sequence and the P_{NFAT}-p53-mCherry encoding cassette. Red fluorescence was detected after ML stimulation in mMscL-expressing cells. (D) Immunoblotting of the cell lysate. The proteins were detected with a 6-His antibody (p53-mCherry) and β-Actin antibody.

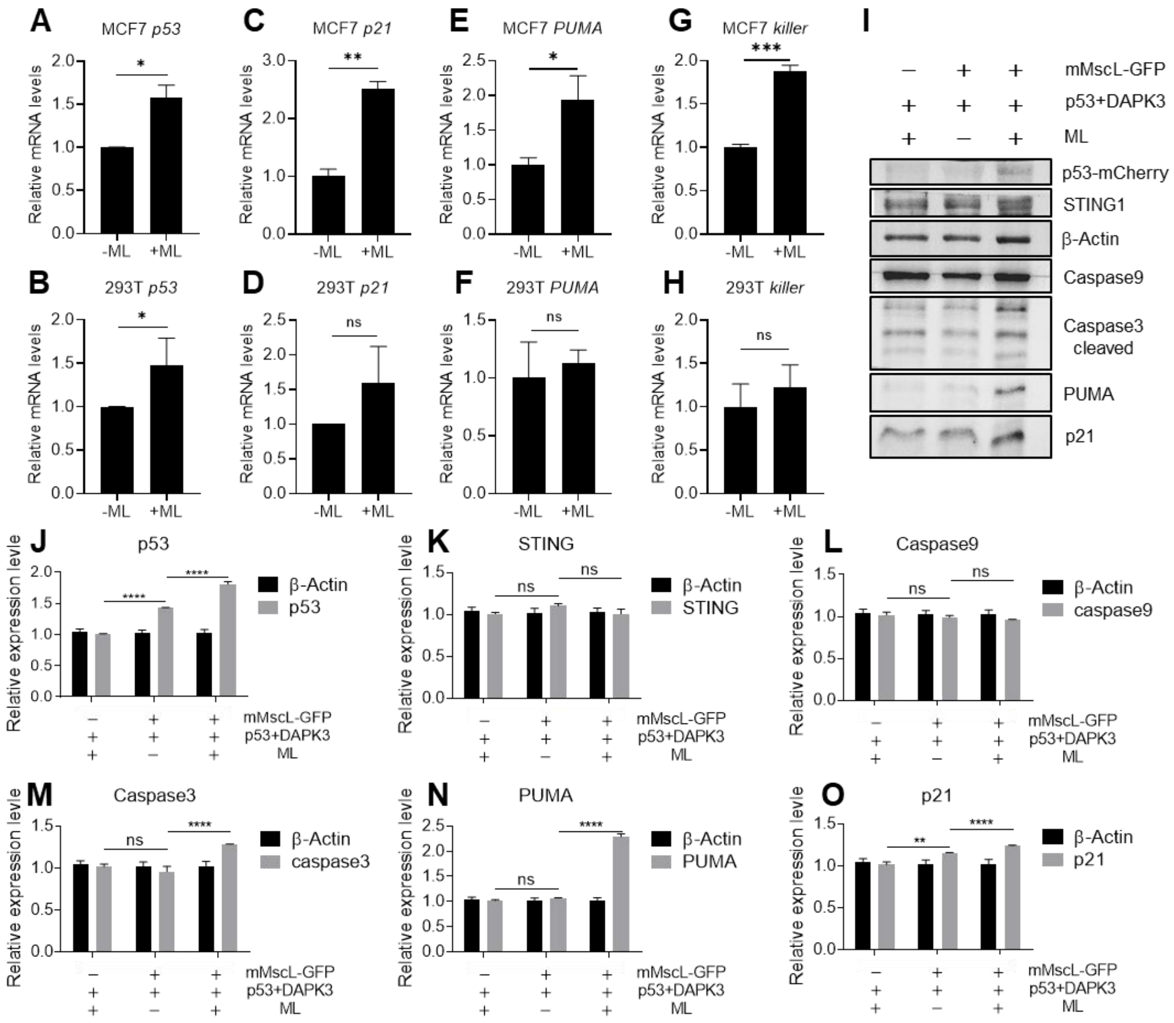

Figure S4. Expression levels of p53 and its regulated proteins controlled by ML stimulation in normal and tumor cells. (A-H) Relative antitumor gene expression levels was detected by RT-PCR. The normal cells (293T) and cancer cells (MCF7) transfected with mMscL, P_{NFAT}-p53-mCherry and P_{NF6}-DAPK3-LUC coding sequence was stimulated by centrifugation for 5 min per hour and were then detected after 12 h of static culture (n=3). (I) Western blots of total cell lysates from transected MCF7 cells. (J-O) The quantitative analysis of Figure S4I. The interested gene p53, DAPK3 and apoptosis-related genes were detected by specific monoclonal antibodies. The error bars indicate the SEMs; ns, no significance; *, $p<0.05$; **, $p<0.01$; and ***, $p<0.001$; two-tailed Student's t test.

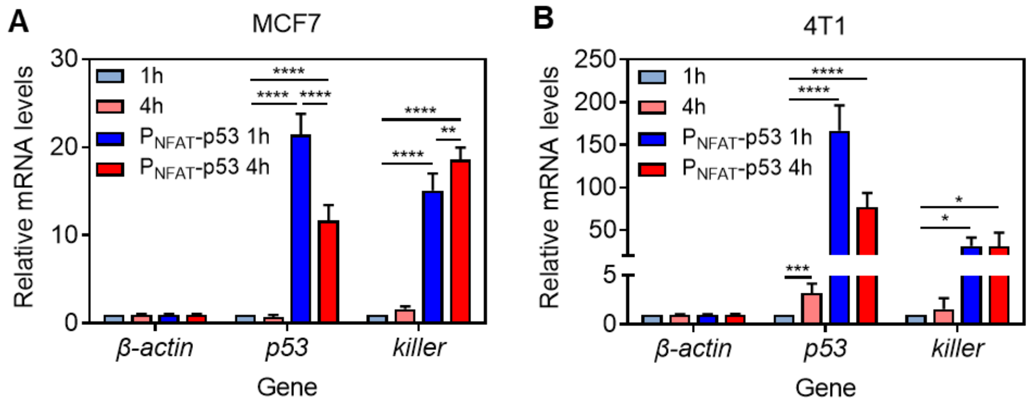

Figure S5. Transcript stability of p53 and killer determined by RT-PCR. (A-B) Relative antitumor gene expression levels in human breast cancer (MCF7) and mouse breast cancer cells (4T1) transfected with mMscL and P_{NFAT}-p53 coding sequence. The cells were stimulated by centrifugation for 5 min per hour and were then detected after 1 h or 4 h of static culture (n=3). The half-life of the p53 transcript in vitro was found to be less than 3 h. The decrease of p53 transcript had no effect on the tumor apoptosis signal, such as killer. The error bars indicate the SEMs; *, $p<0.05$; **, $p<0.01$; and ****, $p<0.0001$; two-tailed Student's t test.

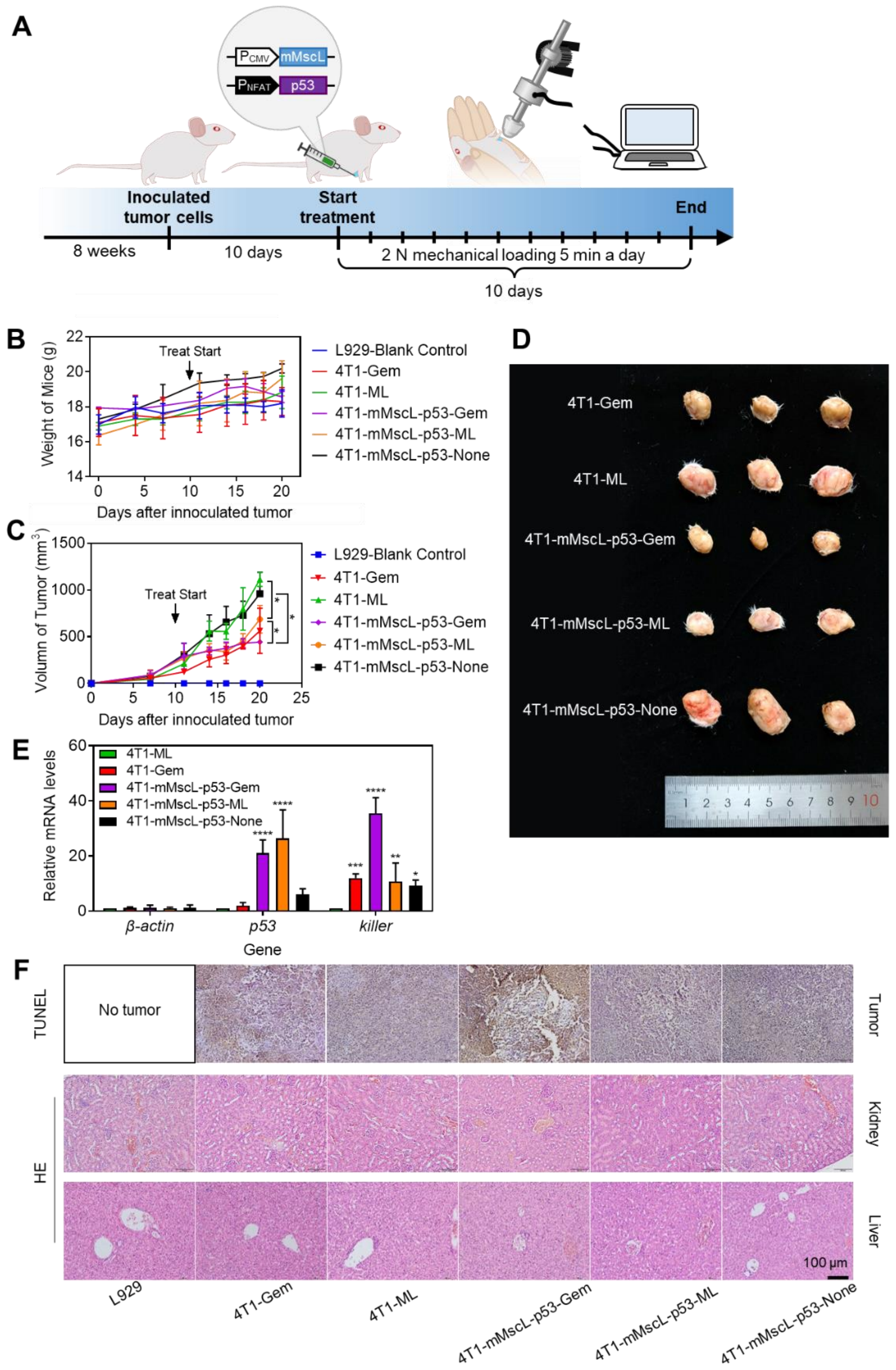

Figure S6 . Tumor-bearing Balb/c mice treated with mechanical loading and gemcitabine. (A) Timeline of the experimental design. Treatment commenced with mechanical force stimulation or gemcitabine gavage administration, once the tumors reached 100 mm^3 in 4T1 tumor mice. The group without transfection was named as "4T1-Gem" (only giving gemcitabine) or "4T1-ML" (only giving mechanical force). The group transfected with P_{CMV}-mMscL+P_{NFAT}-p53 was

named as “4T1-mMscL-p53-Gem” (giving gemcitabine) or “4T1-mMscL-p53-ML” (giving mechanical force). The group transfected with P_{CMV}-mMscL+P_{NFAT}-p53 was named as “4T1-mMscL-p53-None” (without any other treatment). On the 20th day postinoculation, all the mice were euthanized, and their tumors were measured and assessed for apoptosis. (B) Mice weights and (C) tumor volume. The data points and error bars represent the means ±SEMs of 3 mice. (*p<0.05 according to two-tailed Student’s t test.) (D) Photographs of the tumor tissue were taken. The tissues were extracted from the mice under examination on day of 20th after inoculation with tumor cells. (E) The transcription level of the antitumor gene was determined using RT-PCR. Tumor tissues were lysed using liquid nitrogen and the group '4T1-ML' was used as the control group for ΔΔCT method. The error bars indicate the SEMs; *, p<0.05; **, p<0.01; ***, p<0.001; and ****, p<0.0001; two-tailed Student’s t test. (F) After the mice were euthanized at the end of the experiment, the tumors, kidneys, and livers of the tumor-bearing mice were collected and assessed via hematoxylin-eosin (HE) staining and TdT-mediated dUTP-biotin nick end labeling (TUNEL) staining.

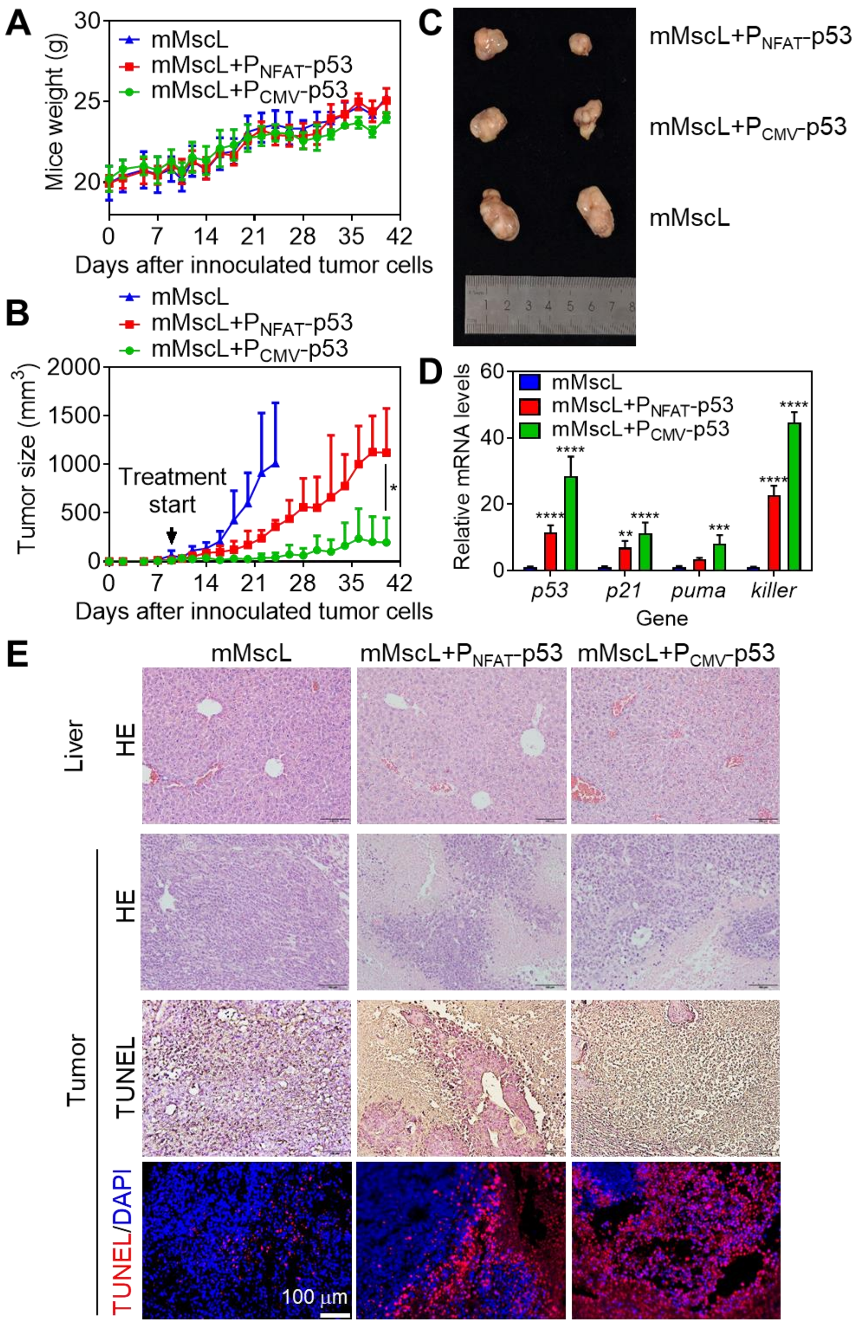

Figure S7. Relationships of tumor-bearing Balb/c-nude mice treated with mechanical loading. (A) Mice weights and (B) tumor volumes of MCF7 tumor model mice were counted when the tumor cells were inoculated (n=5). The treatment timeline was the same as that in Figure S6. (C) Photographs of tumor tissue. The tissue was extracted from the studied mice when its volume reached up to 1500 mm^3 or on day 40 after the tumor cells were inoculated. (D) The transcription level of the antitumor gene was determined using RT-PCR. Tumor tissues were lysed using liquid nitrogen and the group 'mMscL' was used as the control group for ΔΔCT method. The error bars indicate the SEMs; **, p<0.01; ***, p<0.001; and ****, p<0.0001; two-tailed Student's t test. (E) Analysis of HE and TUNEL staining of liver and tumor tissue.

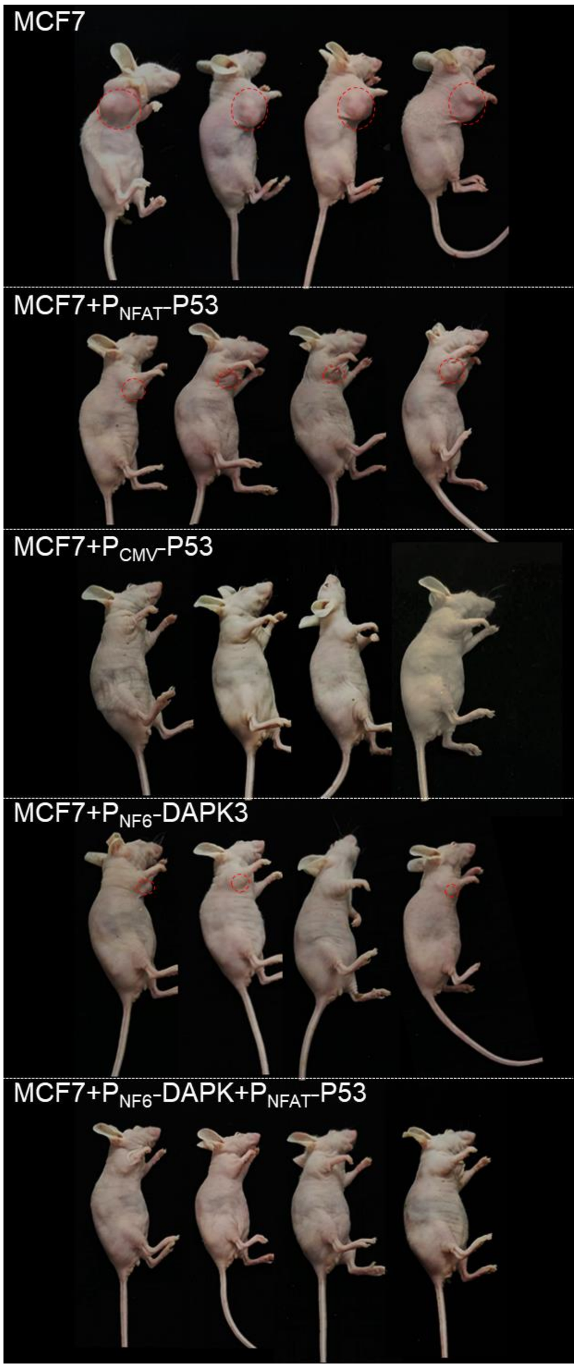

Figure S8. Images of tumor bearing Balb/c-nude mice after daily mechanical treatment.

The images were taken on the 30th day after tumor treatment. The mice were individually transfected with the genes labeled in the images and were treated with 5 min of 2N load pressing per day as shown in Figure 4a.

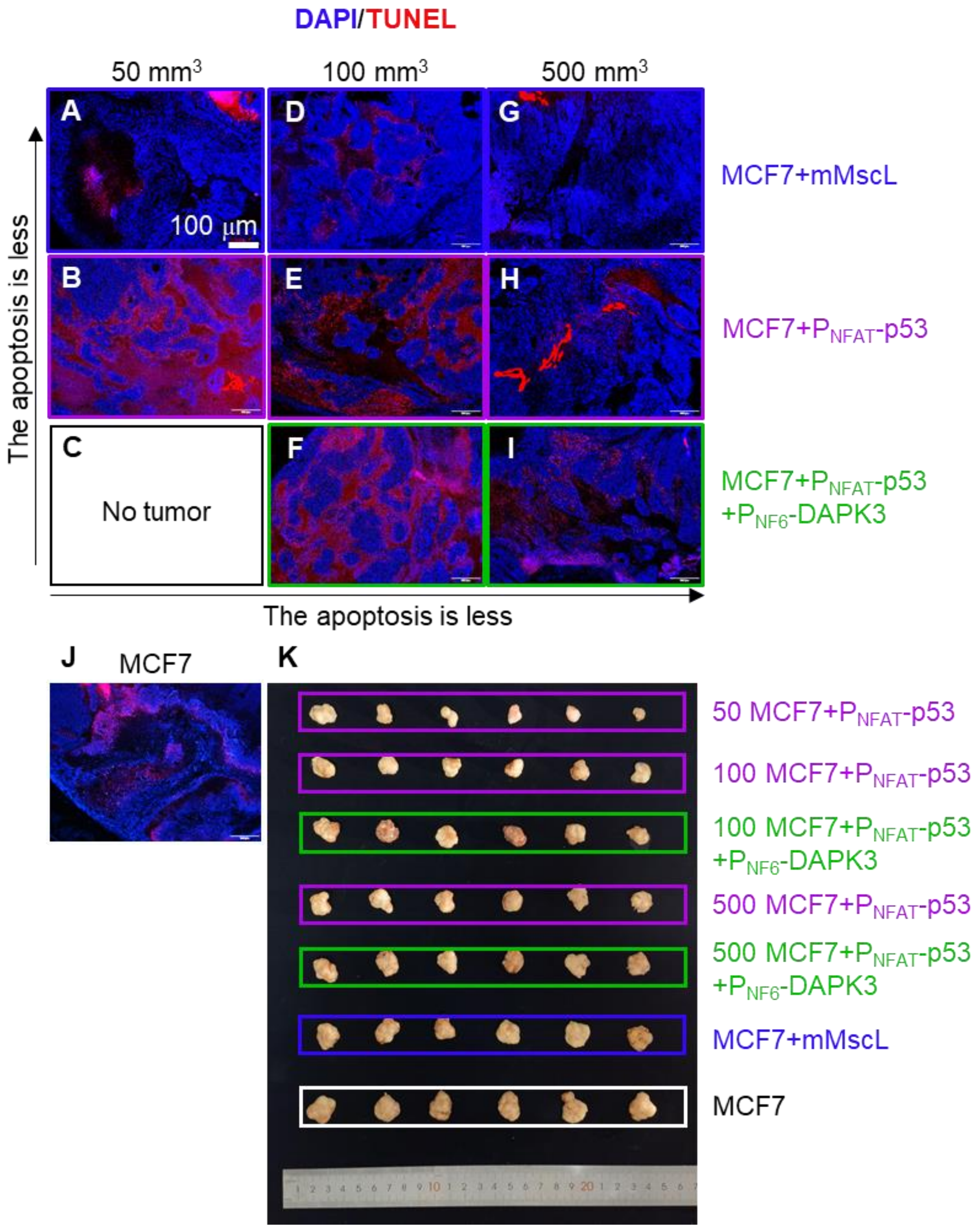

Figure S9. Characteristics of tumor tissues subjected to mechanogenetic pathway transduction in tumors at different stages of development. (A-J) TUNEL staining was carried out on tumor sections following the experiment. (K) Photographs of isolated tumor tissue.

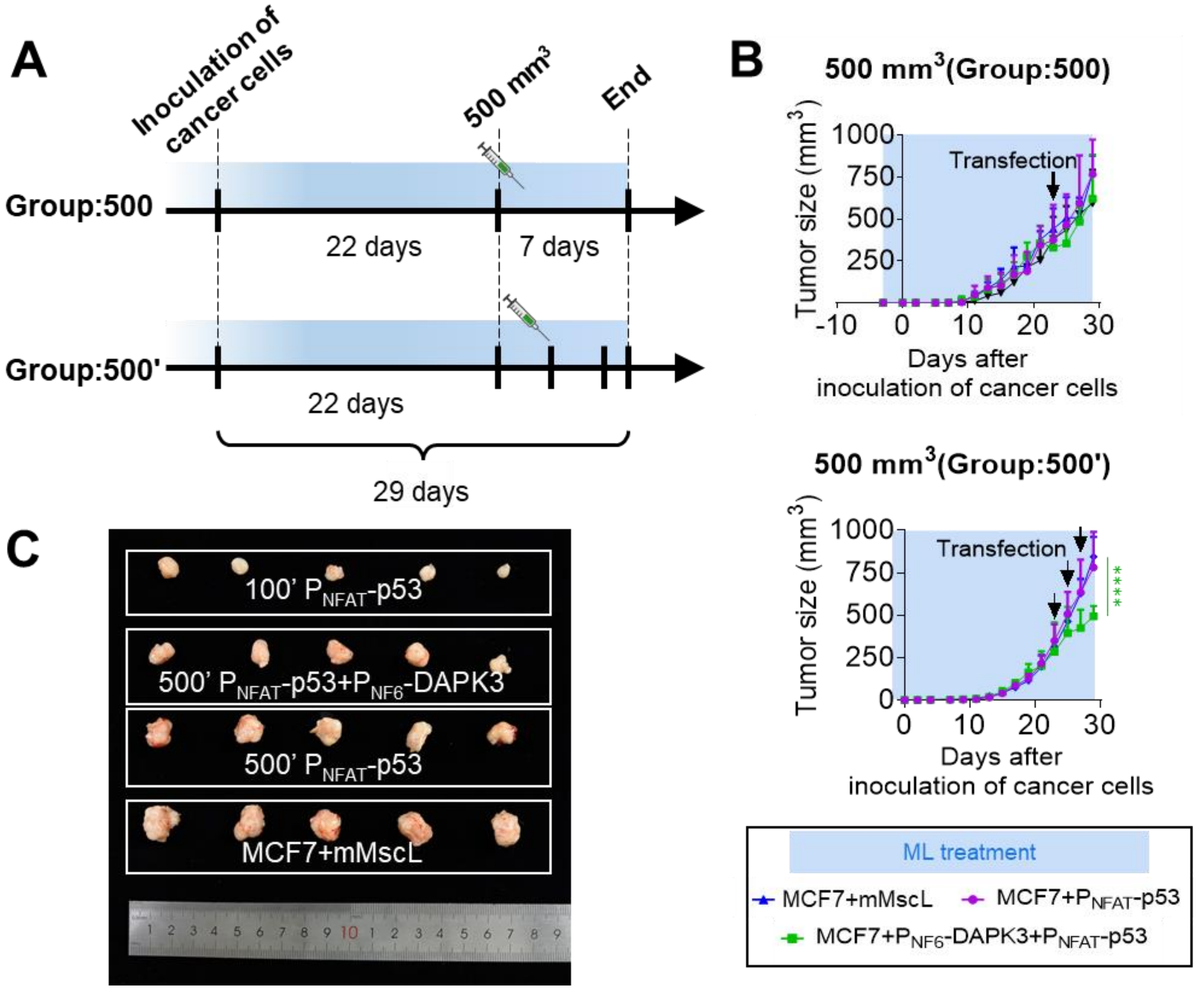

Figure S10. The effects of transfection timing of multiple mechanogenetic circuits on tumors therapy. (A) Schematic diagram of the enhanced experiment for treating mice with 500-mm^3 volume of tumor. To clearly compare the effects of the transfection start period on tumor growth, the beginning of ML was the 3rd day before the inoculation of cancer cells. The group transfected LVs only one time when the volume of tumor tissue reached 500 mm^3, which was named " 500 ". The group transfected LVs three times in 6 days when the tumor size reached 500 mm^3, which was named " 500' ". (B) Tumor growth of mice in multiple transfection experiments (n=5). The black arrow indicated the time point at which LVs were transfected. In group " 500' ", LVs was injected at a therapeutic interval of 1 day for enhancing transformation. (C) Photographs of isolated tumor tissue from Figure 5E and Figure S9B. The error bars indicate the SEMs; ****, p<0.0001; two-tailed Student's t test.

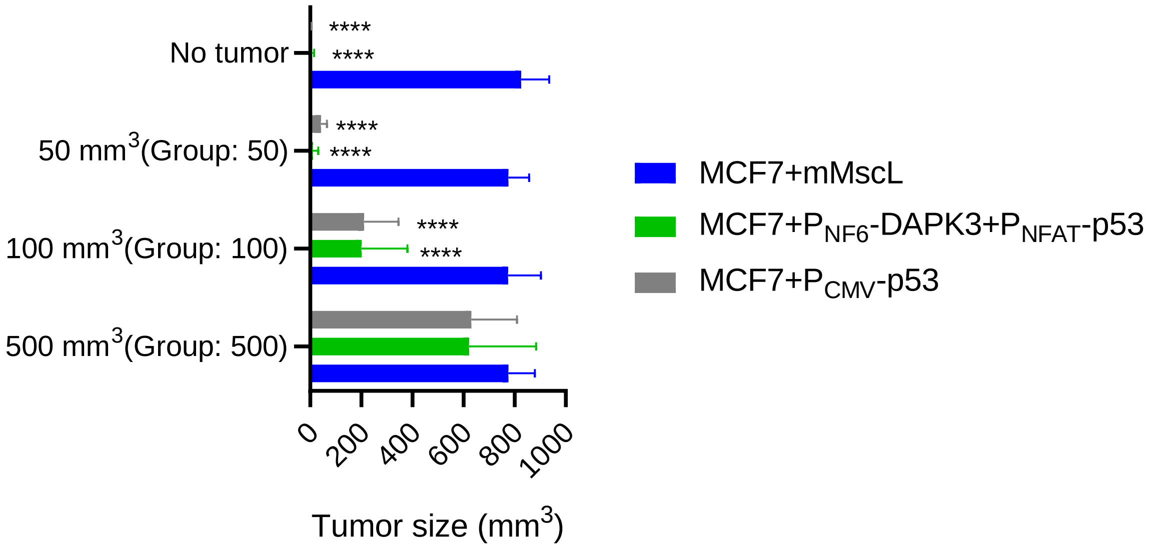

Figure S11. The comparison of tumor sizes for one-time transfection of the mechanogenetic circuits. The nude mice were started to treat by robotic actuation at 3 days before the inoculation of MCF7 cells as shown in Fig 5A and Figure S10A. The mechanogenetic circuits were transfected one time at 3 days before the inoculation of MCF7 ("No tumor"), or 12 days (Group: 50), 17 days (Group: 100), 22 days (Group: 500) after the inoculation of MCF7. The mMscL transfection group (MCF7+mMscL) was the control group for the test. The mMscL and P_{NF6}-DAPK3+P_{NFAT}-p53 simultaneously transfected group was named as "MCF7+ P_{NF6}-DAPK3+P_{NFAT}-p53). The mMscL and P_{CMV}-p53 simultaneously transfected group was named as "MCF7+P_{CMV}-p53". The error bars indicate the SEMs; ****, $p<0.0001$; two-tailed Student's t test.

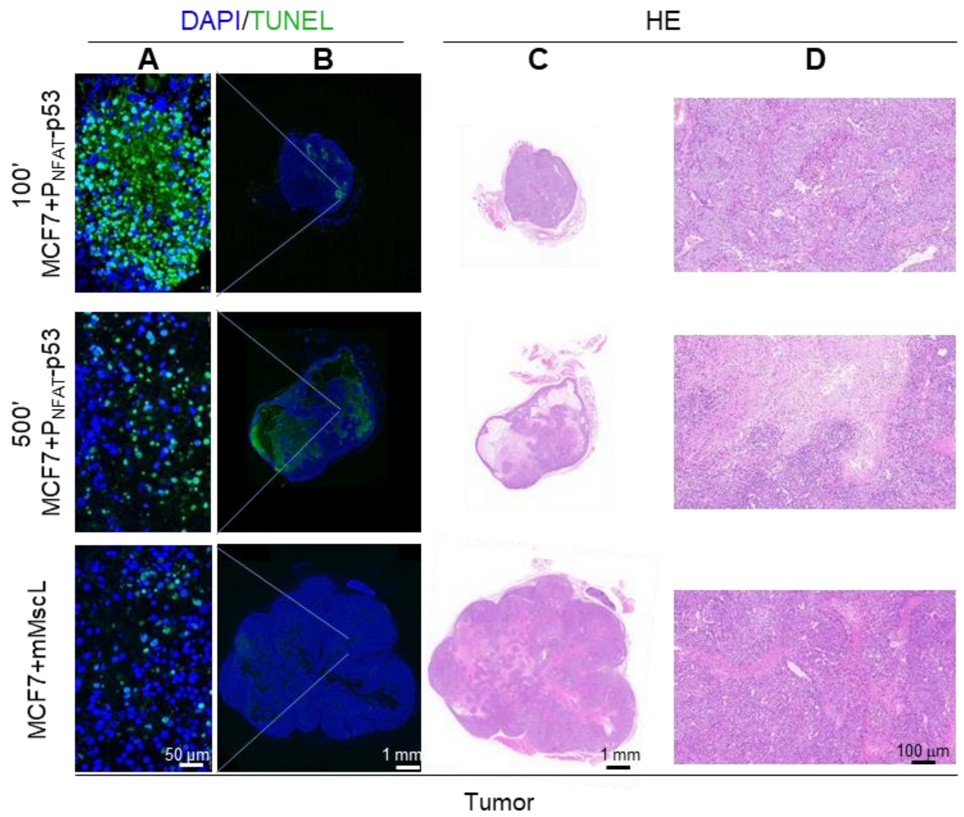

Figure S12. Immunofluorescence and HE staining of tissue sections. (A-B) The tissue from Figure S9c was detected by TUNEL staining. Immunofluorescence was utilized to evaluate the function of the single mechanogenetic circuit transfected into the tumor of group “ 100' ” or “ 500' ”. (C-D) HE staining of the above samples was utilized to observe the morphology of the tumor tissue.

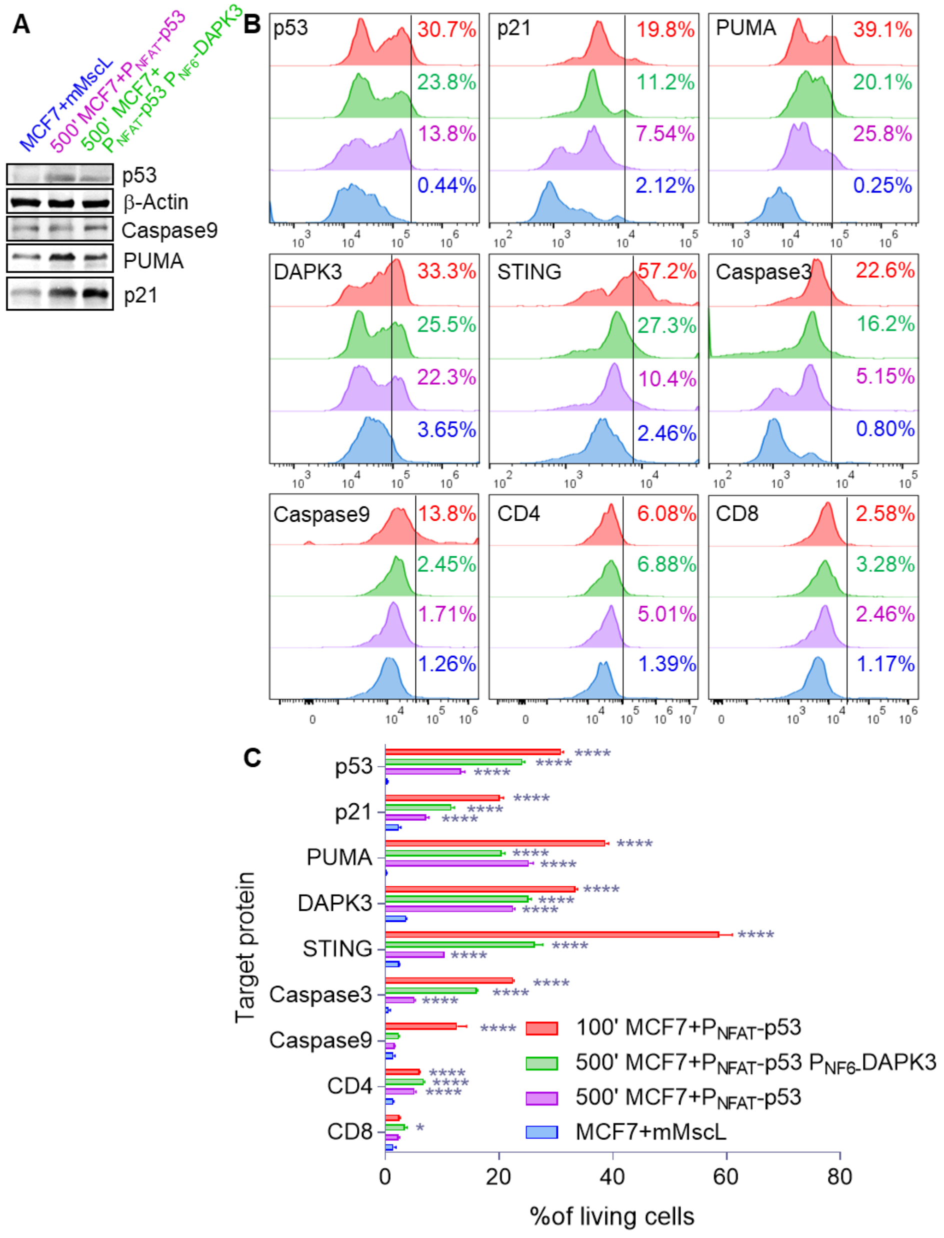

Figure S13. Western blots and flow cytometry of isolated tumor tissues. (A) Western blots of total cell lysates from the tumor tissues shown in Figure S9c. The interested gene p53, DAPK3 and apoptosis-related genes were detected by specific monoclonal antibodies. (B-C) Detection of protein expression by flow cytometry (n=3). The isolated tumor tissues of mice that underwent mechanogenetic therapy are shown in Figure S9C. The error bars indicate the SEMs; ****, p<0.0001; two-tailed Student's t test.

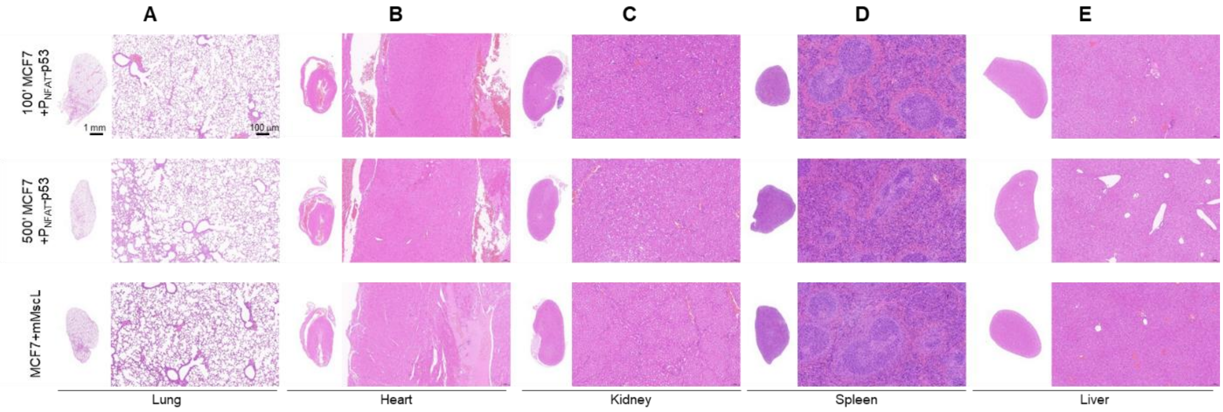

Figure S14. HE staining of tissue organs. The organs were isolated from mice that underwent multiple transfections via a mechanogenetic circuit of group " 100' " or " 500' ".

Table S1. Sequences of the proteins and genes included in the article

Name	Sequence (N' to C' or 5' to 3')	Source
**mMscL**	MSIIKEFREFAMRGNVVDLAVGVIIGAAFGKIVSSLVADI IMPPLGLLIGGIDFKQFAVTLRDAQGDIPAVVMHYGVFI QNVFDFLIVAFAIFMALKLINKLNRKKEEPAAAPAPTKE EVLLTEIRDLLKEQNNRS*	Synthesis referred to Ref[14b, 15b]
**GFP**	MVSKGEELFTGVVPILVELDGDVNGHKFSVSGEGEGD ATYGKLTLKFICTTGKLPVPWPTLVTTLTYGVQCFSRYP DHMKQHDFFKSAMPEGYVQERTIFFKDDGNYKTRAE VKFEGDTLVNRIELKGIDFKEDGNILGHKLEYNYNSHN VYIMADKQKNGIKVNFKIRHNIEDGSVQLADHYQQNT PIGDGPVLLPDNHYLSTQSALSKDPNEKRDHMVLLEFV TAAGITLGMDELYKSGLRSRAQASNSAVDGTAGPGSTG SR*	Synthesis referred to plasmid pEGFP-C1
**mCherry**	MVSKGEEDNMAIIKEFMRFKVHMEGSVNGHEFEIEGE GEGRPYEGTQTAKLKVTKGGPLPFAWDILSPQFMYGS KAYVKHPADIPDYLKLSFPEGFKWERVMNFEDGGVVT VTQDSSLQDGEFIYKVKLRGTNFPSDGPVMQKKTMG WEASSERMYPEDGALKGEIKQRLKLKDGGHYDAEVK TTYKAKKPVQLPGAYNVNIKLDITSHNEDYTIVEQYER AEGRHSTGGMDELYKSGLRSRAQASNSAVDGTAGPGS TGSR*	Synthesis referred to plasmid pmCherry-C1
**p53**	MDDLMLSPDDIEQWFTEDPGPDEAPRMPEAAPPVAPAP AAPTPAAPAPAPSWPLSSSVPSQKTYQGSYGFRLGFLHS GTAKSVTCTYSPALNKMFCQLAKTCPVQLWVDSTPPP GTRVRAMAIYKQSQHMTEVVRRCPHHERCSDSDGLAP PQHLIRVEGNLRVEYLDDRNTFRHSVVVPYEPPEVGSD CTTIHYNYMCNSSCMGGMNRRPILTIITLEDSSGNLLGR NSFEVRVCACPGRDRRTEEENLRKKGEPHHELPPGSTK RALPNNTSSSPQPKKKPLDGEYFTLQIRGRERFEMFREL NEALELKDAQAGKEPGGSRAHSSHSSMHLEGPIL*	Synthesis referred to Ref[3]
**DAPK3**	MSTFRQEDVEDHYEMGEELGSGQFAIVRKCRQKGTGK EYAAKFIKKRRLSSSRRGVSREEIEREVNILREIRHPNIIT LHDIFENKTDVVLILELVSGGELFDFLAEKESLTEDEAT QFLKQILDGVHYLHSKRIAHFDLKPENIMLLDKNVPNP RIKLIDFGIAHKIEAGNEFKNIFGTPEFVAPEIVNYEPLG LEADMWSIGVITYILLSGASPFLGETKQETLTNISAVNY DFDEEYFSNTSELAKDFIRRLLVKDPKRRMTIAQSLEHS WIKAIRRRNVRGEDSGRKPERRRLKTTRLKEYTIKSHS SLPPNNSYADFERFSKVLEEAAAAEEGLRELQRSRRLC HEDVEALAAIYEEKEAWYREESDSLGQDLRRLRQELL KTEALKRQAQEEAKGALLGTSGLKRRFSRLENRYEAL AKQVASEMRFVQDLVRALEQEKLQGVECGLR*	Synthesis referred to Ref[4]
**LUC**	MEDAKNIKKGPAPFYPLEDGTAGEQLHKAMKRYALVP GTIAFTDAHIEVDITYAEYFEMSVRLAEAMKRYGLNTN HRIVVCSENSLQFFMPVLGALFIGVAVAPANDIYNEREL LNSMGISQPTVVFVSKKGLQKILNVQKKLPIIQKIIIMDS KTDYQGFQSMYTFVTSHLPPGFNEYDFVPESFDRDKTI ALIMNSSGSTGLPKGVALPHRTACVRFSHARDPIFGNQI IPDTAILSVVPFHHGFGMFTTLGYLICGFRVVLMYRFEE	Synthesis referred to Ref[5]

	ELFLRSLQDYKIQSALLVPTLFSFFAKSTLIDKYDLSNLH EIASGGAPLSKEVGEAVAKRFHLPGIRQGYGLTETTSAI LITPEGDDKPGAVGKVVPFFEAKVVDLDTGKTLGVNQ RGELCVRGPMIMSGYVNNPEATNALIDKDGWLHSGDI AYWDEDEHFFIVDRLKSLIKYKGYQVAPAELESILLQHP NIFDAGVAGLPDDDAGELPAAVVVLEHGKTMTEKEIV DYVASQVTTAKKLRGGVVFVDEVPKGLTGKLDARKIR EILIKAKKGGKIAV*	
**P_{CMV}**	GACATTGATTATTGACTAGTTATTAATAGTAATCAATTA CGGGGTCATTAGTTCATAGCCCATATATGGAGTTCCGC GTTACATAACTTACGGTAAATGGCCCGCCTGGCTGAC CGCCCAACGACCCCCGCCCATTGACGTCAATAATGAC GTATGTTCCCATAGTAACGCCAATAGGGACTTTCCATT GACGTCAATGGGTGGACTATTTACGGTAAACTGCCCA CTTGGCAGTACATCAAGTGTATCATATGCCAAGTACG CCCCCTATTGACGTCAATGACGGTAAATGGCCCGCCT GGCATTATGCCCAGTACATGACCTTATGGGACTTTCCT ACTTGGCAGTACATCTACGTATTAGTCATCGCTATTAC CATGGTGATGCGGTTTTGGCAGTACATCAATGGGCGT GGATAGCGGTTTGACTCACGGGGATTTCCAAGTCTCC ACCCCATTGACGTCAATGGGAGTTTGTTTTGGCACCA AAATCAACGGGACTTTCCAAAATGTCGTAACAACTC CGCCCCATTGACGCAAATGGGCGGTAGGCGTGTACG GTGGGAGGTCTATATAAGCAGAGCT	Synthesis referred to pEGFP-C1
**P_{NFAT}**	CTAGCTACATTGGAAAATTTTATACACGTTCTAGCTAC ATTGGAAAATTTTATACACGTTCTAGCTACATTGGAA AATTTTATACACGTTCTAGCTACATTGGAAAATTTTAT ACACGTTCTAGCTACATTGGAAAATTTTATACACGTTA GACTCTAGAGGGTATATAATGGAAGCTCGACTTCCAG CTTGGCAATCCGGTACTGTTGGTAAA	Synthesis referred to Ref[6]
**P_{NF6}**	GTCGACCGTGGAGGAAAACTGTTTCATACAGAAGGC GTGGAGGAAAACTGTTTCATACAGAAGGCGTGGAGG AAAACTGTTTCATACAGAAGGCGTGGAGGAAAACTG TTTCATACAGAAGGCGTGGAGGAAAACTGTTTCATAC AGAAGGCGTGGAGGAAAACTGTTTCATACAGAAGGC GTCGCGGAGACTCTAGAGGGTATATAATGGCTT	Synthesis referred to Ref[7]

Table S2. Real-time fluorescent quantitative PCR primers

**Name**	**Nucleotide Sequence (5' to 3')**
GPDH-qF	ACCCACTCCTCCACCTTTGA
GPDH-qR	CTGTTGCTGTAGCCAAATTCGT
β-Actin-qF	GGTCATCACTATTGGCAACG
β-Actin-qR	ACGGATGTCAACGTCACACT
p53-qF	ATGGATGATTTGATGCTGTC
p53-qR	GAGCAGCCTCTGGCATTCTG
p21-qF	CGCGACTGTGATGCGCTAATG
p21-qR	GGAACCTCTCATTCAACCGCC
PUMA-qF	CCTGGAGGGTCCTGTACAATCT
PUMA-qR	GCACCTAATTGGGCTCCATCT
Killer-qF	TGACTCATCTCAGAAATGTCAATTCTTA
Killer-qR	GGACACAAGAAGAAAACCTTAATGC

Table S3. Monoclonal antibodies used for immunoblotting and flow cytometry. The fluorescent antibody used for flow cytometry was obtained with a Lighting-Link Kit (Abacm, USA) and included ab236553 for 488 nm fluorescence and ab269823 for 647 nm fluorescence.

**Name**	**No.**	**Manufactory**	**Host**
p53	bs-2090R	bioss	Rabbit
Anti-6His	bs-0287R	bioss	Rabbit
DAPK3	bs-1692R	bioss	Rabbit
β-Actin	bs-0061R	bioss	Rabbit
STING1	bs-8335R	bioss	Rabbit
Caspase9	bs-3083R	bioss	Rabbit
Caspase3	bs-0081R	bioss	Rabbit
PUMA	bs-1573R	bioss	Rabbit
p21	bs-55160R	bioss	Rabbit
CD4	bsm-52469R	bioss	Rabbit
CD8	bs-10699R	bioss	Rabbit

Movie S1. Fluorescence imaging of calcium influx after stimulation via three different weights.

Movie S2. Tumor-bearing mouse was being treated with mechanical compression.

References

[1] C. Raymond, R. Tom, S. Perret, P. Moussouami, D. L'Abbe, G. St-Laurent, Y. Durocher, *Methods* **2011**, *55* (1), 44.

[2] L. Wang, G. Li, L. Cao, Y. Dong, Y. Wang, S. Wang, Y. Li, X. Guo, Y. Zhang, F. Sun, X. Du, J. Su, Q. Li, X. Peng, K. Shao, W. Zhao, *Sci. Adv.* **2021**, *7* (43), eabj4796.

[3] F. Piras, M. Riba, C. Petrillo, D. Lazarevic, I. Cuccovillo, S. Bartolaccini, E. Stupka, B. Gentner, D. Cittaro, L. Naldini, A. Kajaste-Rudnitski, *EMBO Mol. Med.* **2017**, *9* (9), 1198.

[4] J. Brognard, Y. W. Zhang, L. A. Puto, T. Hunter, *Cancer Res.* **2011**, *71* (8), 3152.

[5] K. Krawczyk, S. Xue, P. Buchmann, G. Charpin-El-Hamri, P. Saxena, M.-D. Hussherr, J. Shao, H. Ye, M. Xie, M. Fussenegger, *Science* **2020**, *368* (6494), 993.

[6] M. Xie, H. Ye, H. Wang, G. Charpin-El Hamri, C. Lormeau, P. Saxena, J. Stelling, M. Fussenegger, *Science* **2016**, *354* (6317), 1296.

[7] E. Merlet, L. Lipskaia, A. Marchand, L. Hadri, N. Mougenot, F. Atassi, L. Liang, S. N. Hatem, R. J. Hajjar, A. M. Lompre, *Gene Ther.* **2013**, *20* (3), 248.
